# Recovery of Phosphorus From Swine Manure by Ultrasound/H_2_O_2_ Digestion, Struvite Crystallization, and Ferric Oxide Hydrate/Biochar Adsorption

**DOI:** 10.3389/fchem.2018.00464

**Published:** 2018-10-08

**Authors:** Tao Zhang, Qiming Wang, Yaxin Deng, Rongfeng Jiang

**Affiliations:** ^1^Beijing Key Laboratory of Farmland Soil Pollution Prevention and Remediation, Key Laboratory of Plant-Soil Interactions of Ministry of Education, Biomass Engineering Center, College of Resources and Environmental Sciences, China Agricultural University, Beijing, China; ^2^Institute for Agricultural Engineering, University of Hohenheim, Stuttgart, Germany; ^3^Illinois Sustainable Technology Center, University of Illinois Urbana-Champaign, Champaign, IL, United States

**Keywords:** swine manure, phosphorus, ultrasound/H_2_O_2_ digestion, struvite, ferric oxide hydrate (HFO)/biochar

## Abstract

Swine manure is potentially harmful to the environment but is also a readily accessible local source of phosphorus (P) for agricultural use. Decreasing the environmental impact of swine manure and recovering P from swine manure have been a challenge for a long time. In this study, an integrated process involving ultrasound/H_2_O_2_ digestion, struvite crystallization, and ferric oxide hydrate (HFO)/biochar adsorption was used to recover P from swine manure. The ultrasound/H_2_O_2_ treatment effectively solubilized the swine manure and converted organic P and other sparingly soluble P species into soluble phosphate. The struvite crystallization process allowed 85% of the available P to be recovered at pH 10.0 using a Mg:P molar ratio of 1.4 and a stirring rate of 150 rpm. HFO was loaded onto biochar synthesized by pyrolyzing ground corncob. The mechanism through which P was adsorbed was investigated by X-ray photoelectron spectroscopy and Fourier transform infrared spectroscopy. The adsorption of P by the HFO/biochar followed pseudo-second-order kinetics and was primarily controlled by chemical processes. The maximum amounts of P adsorbed were 225.08–242.21 mg/g. Thermodynamic calculations indicated that the adsorption of P was endothermic and spontaneous and increased the degree of disorder in the overall system. P mass balance calculations indicated that 90.4% of the total P was recovered as struvite and P-saturated HFO/biochar.

## Introduction

Swine manure has become an important type of agricultural waste in China. Swine manure contains numerous pollutants, including heavy metals, antibiotics, and nutrients. Swine manure that has not been treated in an appropriate way can produce greenhouse gases, cause eutrophication in water bodies, and contaminate groundwater (Jin et al., 2009). Swine manure produced at many pig farms is anaerobically digested (to produce biogas) or composted. However, these biochemical treatments rely on microorganisms, the efficiencies of which are controlled by the temperature, the organic substrates present, and other factors. The use of thermochemical methods (including microwave treatment, hydrothermal processes, pyrolysis, and ultrasonic treatment) for treating swine manure has been investigated (Zhang et al., 2018).

Ultrasonic degradation does not require the presence of exogenous substances and involves only the effects of the ultrasonic field produced (Naddeo et al., 2015). Ultrasonic thermal treatments cause organic matter to dissolve (i.e., to be transferred from the solid phase in slurry to the aqueous phase) and are not limited by the factors that limit bioconversion processes, meaning short digestion times can be used (Khanal et al., 2007). However, treating slurry only using an ultrasonic disintegration treatment is relatively inefficient and consumes large amounts of energy. It has been found in some studies that combining of ultrasonic treatment with the addition various reagents can improve the disintegration efficiency through synergistic effects. H_2_O_2_, an oxidizing agent, has been used as such a reactant because it is very reactive and does not act as a secondary pollutant (Dargahi et al., 2015). Oxidation by H_2_O_2_ destroys the structures of manure solids, releasing water and organic compounds. H_2_O_2_ also oxidizes odorous substances, kills pathogenic bacteria, and degrades antibiotics (Uslu and Balcioglu, 2009; Parker et al., 2012; Srinivasan et al., 2015). During ultrasound/H_2_O_2_ treatment organic substances in swine slurry are digested through both ultrasonic field effects and oxidation by H_2_O_2_, which causes oxyradicals such as -OH and -OOH to form (Appels et al., 2008).

Swine manure has recently started to be regarded as an accessible source of secondary phosphorus (P) for applying to agricultural land (Withers et al., 2015). It is important to recycle P because global phosphate mineral reserves are being depleted and demand for P-based fertilizers is increasing (Elser and Bennett, 2011). Techniques for recover P from secondary resources such as food waste, manure, sewage, and wastewater need to be developed to decrease reliance on P from mineral reserves. Rittmann et al. (2011) found that the livestock industry accounts for 40% of the total loss of P. Recovering P from the livestock sector (e.g., from swine manure) will therefore be an important way of improving the efficiency with which P is used.

Inorganic P in wastewater can be captured directly using single-step P recovery processes such as chemical precipitation, struvite crystallization, adsorption, and ion exchange (Rittmann et al., 2011). For example, crystallizing struvite is a promising way of recovering dissolved P because struvite can be used as a slow-release fertilizer (Jin et al., 2009; Zhang et al., 2014). However, it has been found that struvite can be crystallized from solutions containing very high P concentrations. Several types of dissolved organic matter in swine manure (e.g., veterinary antibiotics, humic acid, and fatty acids) inhibit P recovery using the struvite crystallization method (Lou et al., 2018). Adsorption/desorption processes can recover P from solutions containing low P concentrations and offer several advantages over other methods, being easy to control, requiring minimal resources, and the substrate being able to be recycled. However, adsorption/desorption methods suffer from low capacities and poor selectivity. Research has therefore been focused on developing adsorbents modified with metal oxides (Fang et al., 2015). Adsorbents modified with ferric oxide hydrate (HFO) have recently attracted attention because they can adsorb P very selectively (He et al., 2017). Unfortunately, swine manure contains P in many forms, including as acid soluble organic-P, lipid-P, P in nucleic acids, and sparingly soluble P in orthophosphate and multivalent metal (e.g., aluminum, calcium, iron, or magnesium) cations (He et al., 2017). Integrated multi-step processes will therefore need to be developed to recover P from swine manure.

In this study, an integrated process consisting of ultrasound/H_2_O_2_ digestion, struvite crystallization, and HFO/biochar adsorption for recovering P from swine manure was designed and tested. The ultrasound/H_2_O_2_ treatment solubilized the manure, promoting the conversion of organic phosphorus (OP) and other sparingly soluble P species into soluble inorganic phosphate (IP). The struvite crystallization process was optimized by identifying the most appropriate pH range and Mg:P molar ratio. Finally, the HFO/biochar adsorption process was investigated through assessing surface characterization, adsorption kinetics and adsorption isotherms, and by performing thermodynamic calculations. P mass balance calculations were performed to determine the extraction efficiency.

## Materials and methods

### Materials

Raw swine manure was obtained from a pig farm near Beijing, China. The manure was stored in an ice box until it was used in the experiments. Various parameters for the raw swine manure sample are shown in Table 1.

**Table 1:** Parameters of raw swine manure.

**Items**	**pH**	**Suspended solids**	**Total P**	**${\text{PO}}_{4}^{3-}$-P**	**Available K**	**Dissolved Mg**	**Dissolved Ca**	**Dissolved Fe**	**Dissolved Zn**	**Dissolved Mn**
		**mg**·**L**^−1^								
Parameters	7.2 ± 0.1	6140 ± 211	230.3 ± 7.8	45.5 ± 5.0	111.84 ± 4.42	107.60 ± 2.76	74.91 ± 7.24	0.61 ± 0.14	0.57 ± 0.24	0.13 ± 0.05

The HFO/biochar adsorbent was synthesized by pyrolyzing ground corncob in a cylinder reactor in a muffle furnace at 500°C for 3 h. The cylinder reactor was purged with N_2_ at 10 psi, then placed in the furnace. The reactor and furnace were then purged with N_2_ and sealed to ensure the atmosphere was inert throughout the pyrolysis process. The pyrolyzed material was immersed in 0.8 mol/L FeCl_3(aq)_ at a mass to volume ratio of 1:10, then, 5 mol/L NaOH_(aq)_ was added to adjust the mixture to pH 7. The mixture was then allowed to react for 24 h. The HFO/biochar produced was rinsed with deionized water to remove residual surface ash, dried at 60°C, and sealed in an airtight container until use.

### Methods

#### Ultrasound/H_2_O_2_ digestion

Between 1 and 3.5 mL of 30% H_2_O_2_ was added to 50 mL of the raw swine manure at pH of 3.0, 5.0, or 7.5 at 30 ± 2°C, and each mixture was shaken for 1.5 h. Each mixture was then filtered and the supernatant collected.

#### Struvite crystallization

MgCl_2_ and NH_4_Cl were added to the supernatant of a sample that had been subjected to ultrasound/H_2_O_2_ digestion to give a Mg^2+^:${\text{NH}}_{4}^{+}$:${\text{PO}}_{4}^{3-}$ molar ratio of 0.7–2.1:1:1, then the mixture was adjusted to between pH 5.9 and pH 14.0 and stirred at between 55 and 545 rpm for 2 h at 298 ± 0.5 K. The struvite that crystallized was collected, washed three times with deionized water, and then dried in an oven at 311 K for 48 h.

Response surface methodology (RSM) statistical analysis was performed to assess the relative significance of each experimental factor with regard to P recovery using the struvite crystallization method. The central composite design method was used, and three reaction factors (pH, the Mg/P ratio, and the stirring rate) were evaluated. For such a three variable system the responses could be predicted using the second-order regression equation

(1) $$Y=\beta_{0}+\sum_{i=1}^{3}\beta_{i}X_{i}+\sum_{i=1}^{2}\sum_{j=i+1}^{3}\beta_{ij}X_{i}X_{j}+\sum_{i=1}^{3}\beta_{ii}X_{i}^{2},$$

where Y is the predicted response, β_0_, β_*i*_, β_*ij*_, and β_*ii*_ are the regression coefficients, and *X*_*i*_ and *X*_*j*_ are independent factors. The P recovery efficiency was used as the response in the regression analysis. The analysis was performed using Design Expert version 7.1.6 software (STAT-EASE, Minneapolis, MN, USA).

#### HFO/biochar adsorption

The adsorption kinetics were assessed by mixing 0.075 g samples of HFO/biochar with 20 mL aliquots of the test solution. The mixtures were kept at 293 ± 0.5, 308 ± 0.5, or 323 ± 0.5 K and stirred at 180 rpm. Samples of the supernatants were collected at specific time intervals and each was passed through a 0.45 μm Millipore filter.

Adsorption isotherms were acquired by mixing 0.075 g samples of HFO/biochar with 20 mL aliquots of test samples that had been diluted or to which NaH_2_PO_4_ had been added to give an initial P concentrations of between 10 and 3,000 mg/L. The mixtures were kept at 293 ± 0.5, 308 ± 0.5 or 323 ± 0.5 K and stirred at 180 rpm for 24 h until adsorption equilibrium was achieved.

### Analysis

A swine manure sample was digested using a mixture of H_2_O_2_ and H_2_SO_4_ and then analyzed by inductively coupled plasma-atomic emission spectrometry (ICP-AES; Optima 7300DV, PerkinElmer, USA). The residues and supernatants obtained from the various treatment processes were analyzed by scanning electron microscopy (SEM, S-5400, Hitachi, Japan) and three-dimensional excitation emission matrix fluorescence spectroscopy (3D-EEM, F-7000, Hitachi, Japan).

The specimens that were analyzed in this manner included (a) raw swine manure samples, (b) swine manure samples treated with H_2_O_2_ (2.5 mL 30% H_2_O_2_), (c) swine manure samples treated only by ultrasound (303 K, 1.5 h, 100% power), and (d) swine manure samples at pH 3.0 treated with ultrasound/H_2_O_2_ (303 K, 1.5 h, 100% power, 2.5 mL 30% H_2_O_2_). The 3D-EEM data were analyzed using the parallel factor analysis (PARAFAC) method. An excision-interpolation technique was used to eliminate Rayleigh and Raman scattering peaks before PARAFAC modeling was performed. A nonnegativity constraint was applied to the parameters to allow only chemically relevant results to be used, and PARAFAC models with two to six components were used. The PARAFAC models were fitted to the data using MATLAB 2017a software, using the N-way toolbox.

The struvite samples were analyzed by field emission scanning electron microscopy with energy dispersive X-ray spectrometry using a Merlin Compact instrument (SEM-EDX, XFlash 6100, Bruker, Karlsruhe, Germany), and by X-ray diffraction using a Dmax/2400 instrument (XRD, Rigaku, Tokyo, Japan).

The HFO/biochar and biochar samples were analyzed by Fourier transform infrared spectroscopy (FTIR; Magna-IR 750, Washington, USA), X-ray photoelectron spectroscopy (XPS, Axis Ultra, Shimadzu, Japan), and XRD (Dmax/2400, Rigaku, Japan) to investigate the adsorption mechanism and SEM-EDX (XFlash 6100, Bruker, Karlsruhe, Germany) to determine the surface morphologies.

Each supernatant sample was passed through a filter and analyzed immediately after being collected. The ${\text{PO}}_{4}^{3-}$ concentration in each supernatant sample was determined following the standard method (APHA, 2012, 4500-P C, vanadomolybdophosphoric acid colorimetric method). Each experiment was repeated three times and the mean results for the triplicate experiments are reported.

## Results and discussion

### Ultrasound/H_2_O_2_ digestion

#### Scanning electron microscopy

**Figure 1:**
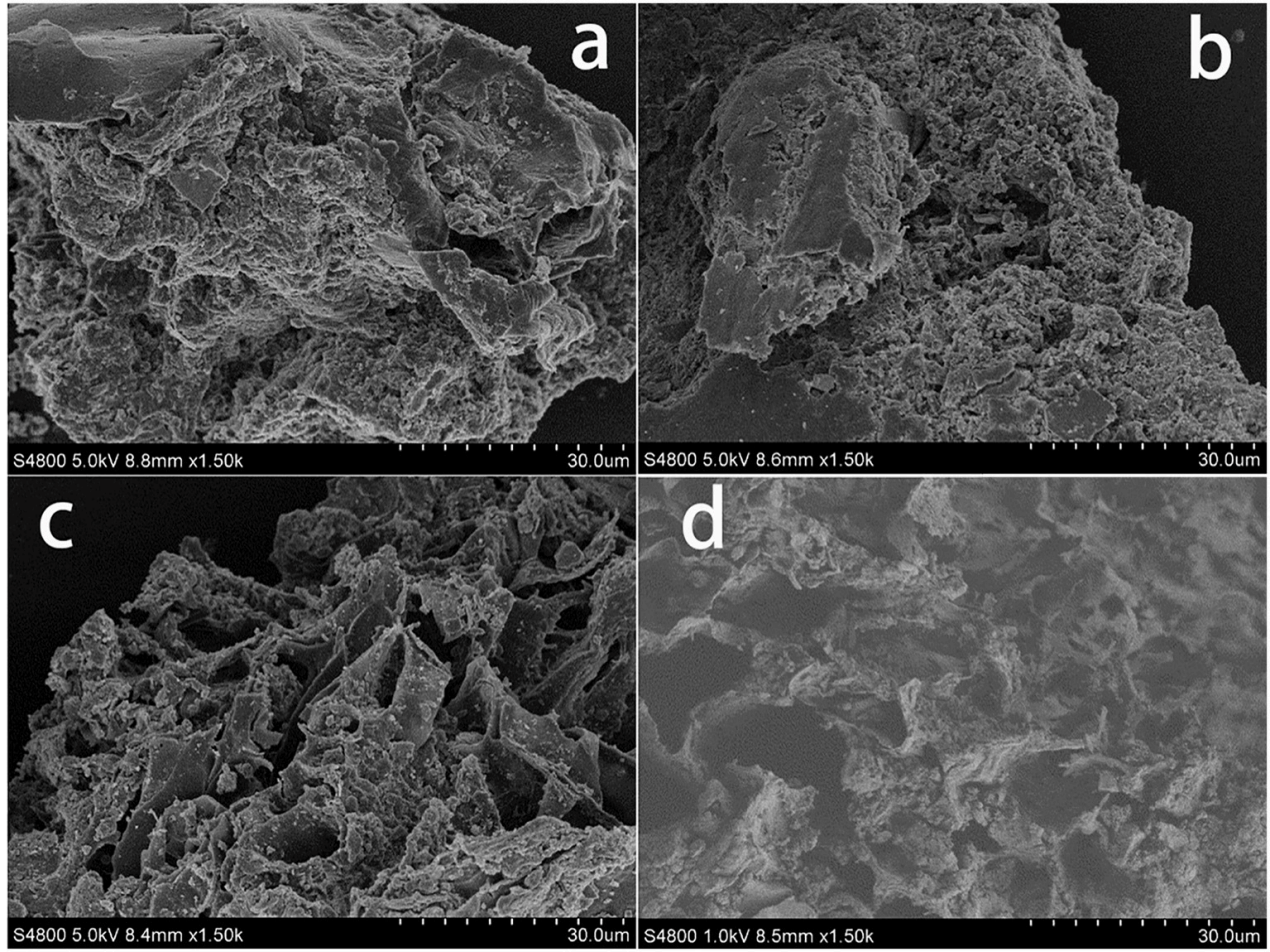
SEM analysis of swine manure residues at different treated processes **(a)** untreated, **(b)** treated with H_2_O_2_ (2.5 mL 30% H_2_O_2_), **(c)** treated solely with ultrasound (303 K, 1.5 h, 100% power), and **(d)** treated with ultrasound/H_2_O_2_ digestion at pH 3.0 (303 K, 1.5 h, 100% power; 2.5 mL 30% H_2_O_2_).

Swine manure residues were analyzed by SEM after the different treatments had been performed, and SEM images of the residues are shown in Figure 1. It can be seen from Figures 1a,b that the raw swine manure residue was closely packed but that adding H_2_O_2_ somewhat loosened the structure. Ultrasound treatment clearly caused the structure to break up more than did H_2_O_2_, as can be seen from the more porous structure shown in Figure 1c. The combined ultrasound/H_2_O_2_ digestion (Figure 1d) resulted in greater collapse and erosion of the material. These results indicated that combing ultrasound and H_2_O_2_ treatments effectively broke down the solid material in the manure sample, solubilizing the P.

#### Three-dimensional excitation emission matrix fluorescence spectroscopy

The 3D-EEM technique was used to analyze the soluble organic compounds in the supernatants produced by the different treatments. Contour EEM spectra of the water-soluble organic matter (WSOM) are shown in Figure S1. It can be seen that different fluorophores were present, each characterized by an excitation/emission wavelength pair. The PARAFAC technique was used to analyze the contour EEM spectra to obtain chemically meaningful components. The results were not conclusive but appeared to show that various biomolecules, such humic and fulvic substances and peptides, were present.

The WSOM obtained from the samples was examined using PARAFAC models with two to seven components. Underspecifying such a model will cause fewer components to be used than there are independently varying chemical moieties responsible for the signal (Murphy et al., 2013). Considering the characteristics of the residual plots and spectral loadings, a four-component model was selected as the most appropriate multiway data model for describing the data set. No consistent patterns were found in the residuals plots for the samples generated using the model (Figure S2), indicating that the residuals were random, as required, although there were with minor peaks along the diagonal. The spectral loadings of the four components showed typical characteristics of independent non-interacting organic fluorophores, as shown in Figure S3.

The excitation and emission spectral loadings for the four components are shown in Figure S3. Chen et al. (2003) quantitatively analyzed of EEM spectra by delineating the EEM landscape into five regions and calculating the integrated volume under each region to allow the dissolved organic matter (DOM) to be characterized. These regions corresponded to aromatic proteins (two regions), fulvic acid, microbial byproducts, and humic acid. The PARAFAC-derived components were therefore considered to be: peak 1 (excitation and emission wavelengths 220 and 350 nm, respectively) associated with aromatic protein II; peak 2 (220/300 nm) associated with aromatic protein I; peak 3 (300/410 nm) associated with humic acid; and peak 4 (280/300 nm) associated with microbial by-products.

It can be seen from Figure S2 that the fluorescence intensity (FI) values of the swine manure supernatant components were different when different treatments had been performed. Compared with raw swine manure (Figure S2a'), the FI of the WSOM in the sample treated with ultrasound was greatly increased, especially that of the soluble proteins (Figure S2c'). The effects of sonication on organic matter solubilization have been described previously. Jiang et al. (2014b) found that the soluble protein concentrations in food waste were increased by sonication and Ruiz-Hernando et al. (2015) found that ultrasound irradiation increased the solubilized protein concentrations in activated sludge. Organic matter solubilization occurs as a result of floc disintegration and cell lysis due to thermal effects, strong shear forces and the oxidative effects of radicals formed during sonication, which was confirmed by the SEM images (Figure 1). The residues after ultrasound treatment showed looser structures and the formation of pores on the particle surfaces (Figure 1c). The ultrasound treated samples had high solubilized organic matter concentrations, meaning that the treatment efficiently made insoluble material soluble and that the oxidation process did not degrade the organic matter.

On the contrary, the FI of the WSOM in the manure treated with H_2_O_2_ was lower than that obtained from the raw material (Figure S2b'), meaning that the soluble protein (aromatic protein II) concentration was decreased strongly by the H_2_O_2_ treatment. This could be explained in terms of radical reaction mechanisms, because H_2_O_2_ can produce numerous hydroxyl radicals capable of oxidizing organic matter to CO_2_ and water (Malgorzata et al., 2018).

The combined ultrasound/H_2_O_2_ treatment was more efficient than the ultrasound only and H_2_O_2_ only treatments in terms organic matter degradation, as shown in Figure S2d'. The fluorescence intensities of all the components were decreased by the combined ultrasound/H_2_O_2_ treatment, and aromatic protein II appeared to be completely eliminated. It has been found that ultrasonic irradiation accelerates H_2_O_2_ dissociation, releasing more free radicals than would otherwise be released (Kobayashi et al., 2014). The free radicals released will promote the oxidation of organic matter. The increases in the pore sizes shown in the SEM images provide evidence for this process (Figure 1d). The mechanical action of sonication was therefore strengthened by the presence of H_2_O_2_. Based on these results, we concluded that during the ultrasound/H_2_O_2_ treatment organic matter degradation proceeded via ultrasonic destruction of the organic material such that polymeric organic components were converted into lower molecular weight substances and soluble organic matter. Free radicals formed through the action of ultrasound on H_2_O_2_ then transformed WSOM into CO_2_ and water.

#### Solubilization of P

**Figure 2:**
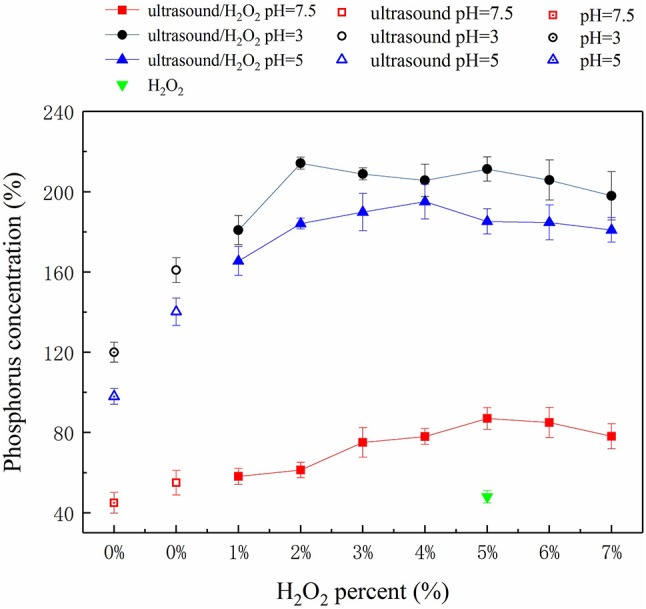
Phosphorus concentration at different treatment processes.

The amounts of P solubilized and released by the ultrasound, H_2_O_2_, and ultrasound/H_2_O_2_ treatments are summarized in Figure 2. The ultrasound/H_2_O_2_ process was the most effective method for solubilizing P, so the amount of H_2_O_2_ added and the pH were optimized for this treatment. The effect of the pH was investigated using acidic manure (at pH 3 and pH 5) and raw swine manure (at pH 7.5, i.e., close to neutral). The results showed that the degree of P solubilization and release efficiency initially increased and then decreased as the amount of H_2_O_2_ added increased. The optimal IP: total phosphorus (TP) ratio was 91.8% at pH 3 and when the volume of H_2_O_2_ added was 5% v/v of the sample volume. Some researcher has reported relevant studies. Kim and Kim (2016) found, in thermal treatment of sludge at 50–80°C, inorganic phosphate release was 19–22% of the total phosphorus in the sludge.

The ultrasonic cavitation effect can cause macromolecular organic compounds (including OP polymers) to change from being flocculated to being dispersed (Gong et al., 2015). Ultrasonic cavitation destabilizes the internal structures of OP macromolecules and weakens some chemical bonds, so free radicals such as •OH and •OOH (decomposition products of H_2_O_2_) can react with the organic compounds and destroy OP functional groups and therefore decompose OP compounds to IP (Pehkonen and Zhang, 2002; Gifford et al., 2015). This process can be summarized as a three-step mechanism. The first step is acid hydrolysis of soluble OP and acid dissolution of sparingly soluble P. Under acidic conditions (pH 3), sparingly soluble P species (such as calcium phosphate and magnesium phosphate) and soluble OP can be dissolved and hydrolyzed. High Ca and Mg concentrations were found in the samples (Table 1), indicating that sparingly soluble P species were responsible for an important proportion of the P released to the liquid phase under acidic conditions. The second step is the ultrasonic cavitation effect causing the OP macromolecules to change from being flocculated to being highly dispersed (Gong et al., 2015). The alternating positive and negative ultrasonic pressure applied causes micro-vesicles occurs to rapidly shrink, rupture, and collapse, and the sharply increasing temperatures of the air bubbles produced creates a gas-liquid interface that contributes to the cavitation effect (Jiang et al., 2014a). The OP in swine manure is primarily present as phospholipids, DNA, simple phosphate monoesters, and phytic acids (Dai et al., 2015; Ekpo et al., 2016a,b). Ultrasonic cavitation disrupts the bonding forces in these OP species and also promotes the generation of free radicals such as •OH (Zhang K. et al., 2011). OP at the gas-liquid interface generated during ultrasonic treatment is readily digested and decomposed by these radicals. In the third step, functional groups in the OP species are cracked, digested, and decomposed. Ultrasonic cavitation has been found to decompose H_2_O_2_ to produce more effective free radicals (Gogate and Pandit, 2004) such as •OH and •OOH (Liu et al., 2013) than would be produced otherwise. These radicals can attack OP, weakening bonds and further breaking down functional groups, leading to oxidative decomposition. Ultrasound therefore plays a dual role in driving and catalyzing the reactions. Ultrasound directly decomposes organic molecules as a driving force of reactions, but as a catalyst ultrasound promotes free oxyradical formation and facilitates oxidative decomposition of organic compounds (Zhang K. et al., 2011). H_2_O_2_ acts as both a source and scavenger of •OH. At low H_2_O_2_ concentrations, little •OH will be generated and the overall digestion rate will be low, but increasing the H_2_O_2_ concentration, causes more •OH radicals to be generated and the digestion rate to increase. However, above a certain H_2_O_2_ concentration, the extra H_2_O_2_ will react with •OH to form •OOH radicals and will therefore compete with organic matter in the manure for •OH. The •OOH radical has a lower activity than •OH, so the consumption of •OH to form •OOH will decrease the digestion rate (Zhang K. et al., 2011; Liu et al., 2013). It can be seen from Figure 2 that the optimum amount of H_2_O_2_ should be added to achieve the best results in terms of releasing P.

The digestion rate of the ultrasound/H_2_O_2_ process was higher under acidic conditions than neutral conditions, and this was attributed to three effects. First, sparingly soluble P species, such as calcium phosphate and magnesium phosphate, tend to dissolve under acidic conditions. Second, organic matter will be present as neutral molecules at lower pH values, meaning that the organic matter will readily access the gas-liquid interfaces of cavitation bubbles. The organic matter could then be thermally decomposed at the interfaces and be involved in free radical reactions within the cavitation air bubbles. In contrast, ionic organic matter would not be able to access the interfaces and disperse within the air bubbles. The decomposition of this organic matter will primarily proceed through reactions with free radicals. Third, increasing the pH, will the H_2_O_2_ self-decomposition rate (Liu et al., 2013). Moreover, ${\text{HO}}_{2}^{-}$ is generated by the deprotonation of H_2_O_2_ and consumes •OH in the solution under alkaline conditions (Jiraroj et al., 2006). This waste of H_2_O_2_ will decrease the oxidative decomposition efficiency. Although acidic conditions can enhance the digestion effect, the rate at which •OH is generated from H_2_O_2_ and the free radical reaction efficiency will decrease if the pH is too low. pH 3 was found to be most appropriate for the ultrasound/H_2_O_2_ digestion process.

### Struvite crystallization

#### Response surface methodology analysis

Zhang and Chen (2009) used RSM to optimize the conditions for recovering ammonium and P from a fermented waste-activated sludge mixture. Zhang T. et al. (2011) used RSM to investigate struvite crystallization from wastewater. We used RSM to improve our understanding of the relative significances of and interactive effects between various reaction factors during struvite crystallization.

The struvite crystallization model designed using the central composite design RSM process and the actual and predicted P recovery efficiency values (Y) are shown in Table S1. Three factors, X_1_-pH, X_2_-Mg:P and X_3_-rotation rate, were assessed, by performing a regression analysis on the experimental data using Design Expert software. The regression analysis results suggested that a quadratic model was the most appropriate for predicting the P recovery efficiency. The quadratic model was shown to be appropriate using an analysis of variance procedure. The analysis of variance results for the quadratic model of the P recovery efficiency are shown in Table S2. The model gave an *F*-value of 7.89 with a very low *P*-value of 0.0017 and an *R*^2^ value of 0.8766, demonstrating that the correlation between the modeled data and the actual data was statistically significant. The P recovery efficiencies predicted by the quadratic model were similar to the experimental values (Table S1). These results indicated that the quadratic model based on RSM analysis was accurate and applicable to struvite crystallization. The equation used to predict the P recovery efficiency was.

(2) $$\begin{array}{l} Y=0.83+0.076X_{1}+0.097X_{2}-0.037X_{3}+0.0056X_{1}X_{2}\\ \;+0.079X_{1}X_{3}+0.027X_{2}X_{3}-0.21X_{1}^{2}-0.11X_{2}^{2}-0.025X_{3}^{2} \end{array}$$

“Prob > F” values generated by the regression analysis of less than 0.05 indicate that the relevant model terms are significant. The data shown in Table S2 indicated that pH and Mg:P gave significant linear correlations and pH × pH and Mg/P × Mg/P gave significant quadratic correlations. These results indicated that the pH and Mg:P ratio were the most important factors that affected the P recovery efficiency.

**Figure 3:**
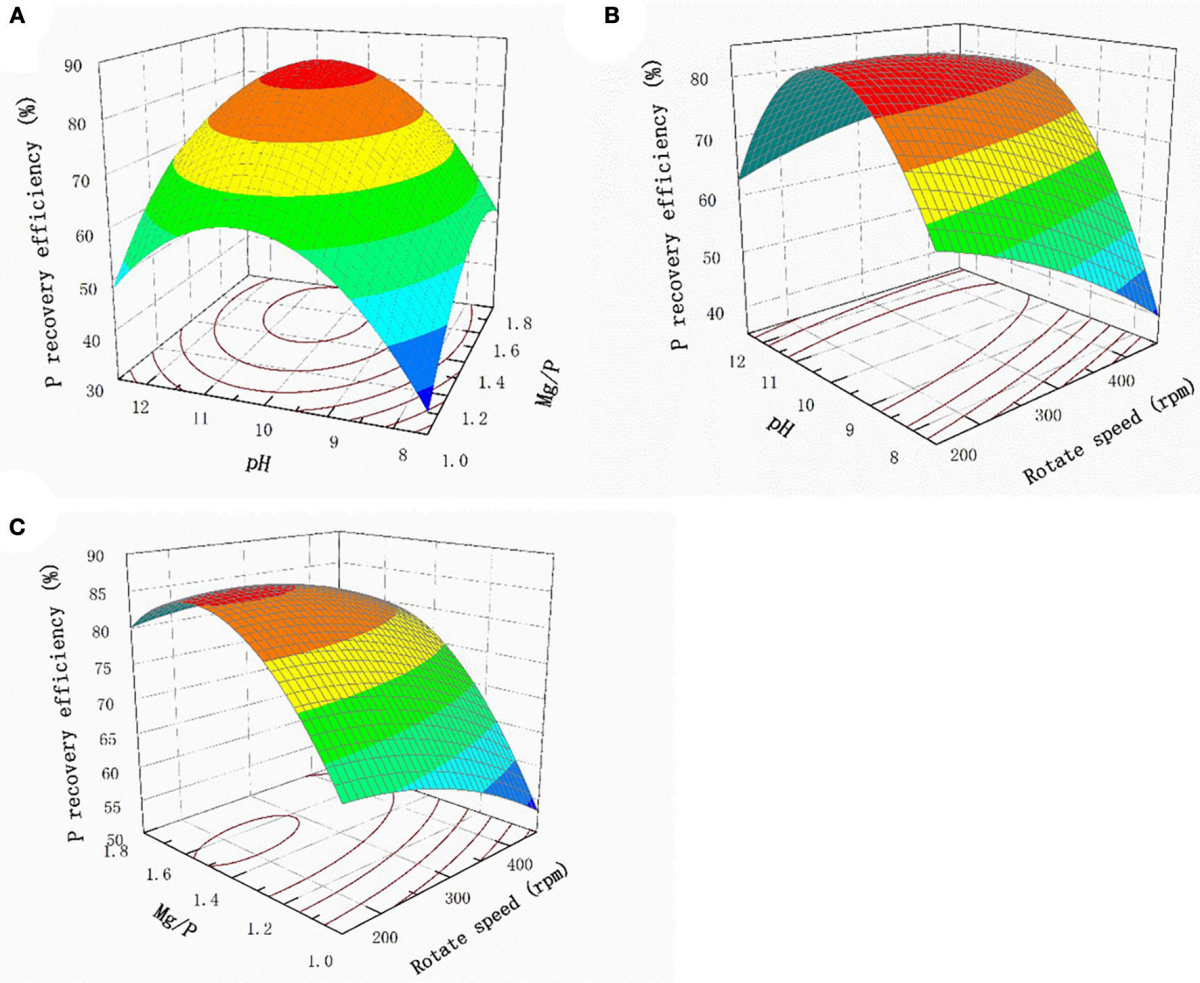
Response surface plots of the interaction effect of different factors on P recovery efficiency **(A)** pH and Mg/P **(B)** pH and rotate speed **(C)** Mg/P and rotate speed.

The response surface plots for the interaction effects of the different factors with regard to the P recovery efficiency are shown in Figure 3. The P recovery efficiency clearly increased as the pH increased when the Mg:P ratio remained constant, and the most efficient recovery was found at pH 10.0, above which the recovery gradually decreased (Figure 3A). The solution pH should therefore be kept in the range pH 9.5–10.5 to achieve optimum efficiency. This was not unexpected, because struvite crystals generally form under alkaline conditions. Similarly, when the pH remained constant, the P recovery efficiency increased as the Mg:P ratio increased from 1 to 1.4. However, when the Mg:P ratio is higher than the optimum value, the Mg ions more likely react with hydroxide ions, causing Mg(OH)_2_ to precipitate. This will inhibit struvite crystallization and slightly decrease the P recovery efficiency. The relationship between the pH and stirring rate at a Mg:P ratio of 1.4 is shown in Figure 3B. It can be seen that the P recovery efficiency remained unchanged as the stirring rate changed, demonstrating that the stirring rate did not markedly affect struvite crystallization. Mass transfer would have been increased by agitation, but the effect of mass transfer during struvite crystallization was clearly weaker than the effect of the pH. The relationship between the Mg/P ratio and the stirring rate at pH 10.0 is shown in Figure 3C. When the Mg:P ratio was constant, the stirring rate only weakly affected struvite crystallization.

#### Struvite characterization

Struvite has a rhombic structure and can crystallize in two main crystal forms: a distinctive orthorhombic structure and needle-like structures of different thicknesses and lengths. The SEM-EDX and XRD results for precipitates produced at pH 10.0 and a Mg:P ratio of 1.4 are shown in Figure S4. The SEM images (Figure S4a) confirmed that the crystals were long needles with struvite structures. Elemental mapping (Figures S4b–d) showed that O, P, and Mg were the three main elements in the crystals.

The composition of the struvite precipitate was further examined by XRD (Figure S5). The pattern for the original residue (captured by a 0.45 μm filter) suggested the struvite was amorphous, but the struvite spectrum matched the PDF card for a struvite standard (JCPDS 15-0762) well, confirming that the precipitation process generated struvite.

### HFO/biochar adsorption

#### Surface characterization

**Figure 4:**
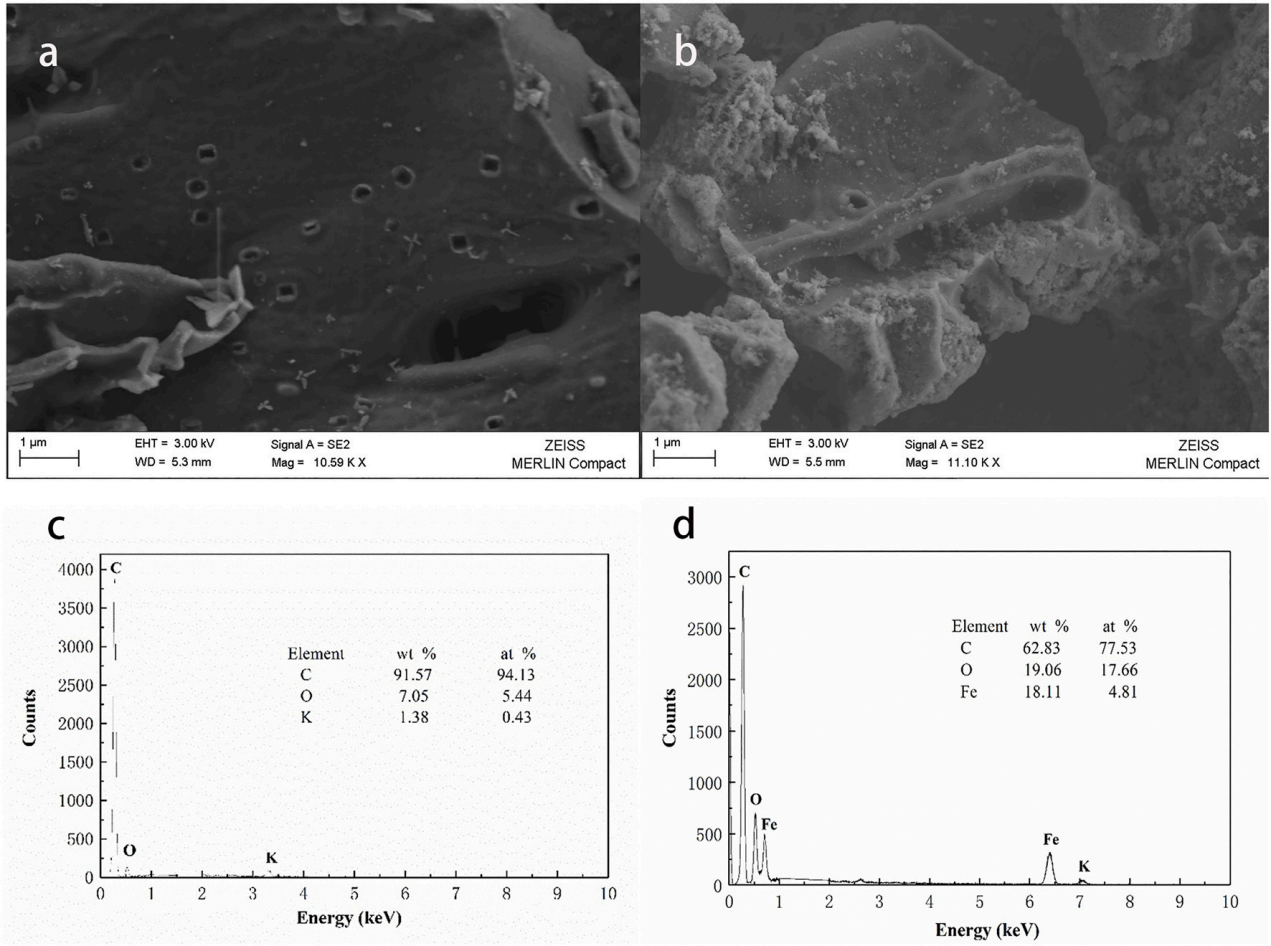
SEM-EDX analysis of biochar and HFO/biochar **(a)** SEM analysis of biochar **(b)** SEM analysis of HFO/biochar **(c)** EDX analysis of biochar **(d)** EDX analysis of HFO/biochar.

**Figure 5:**
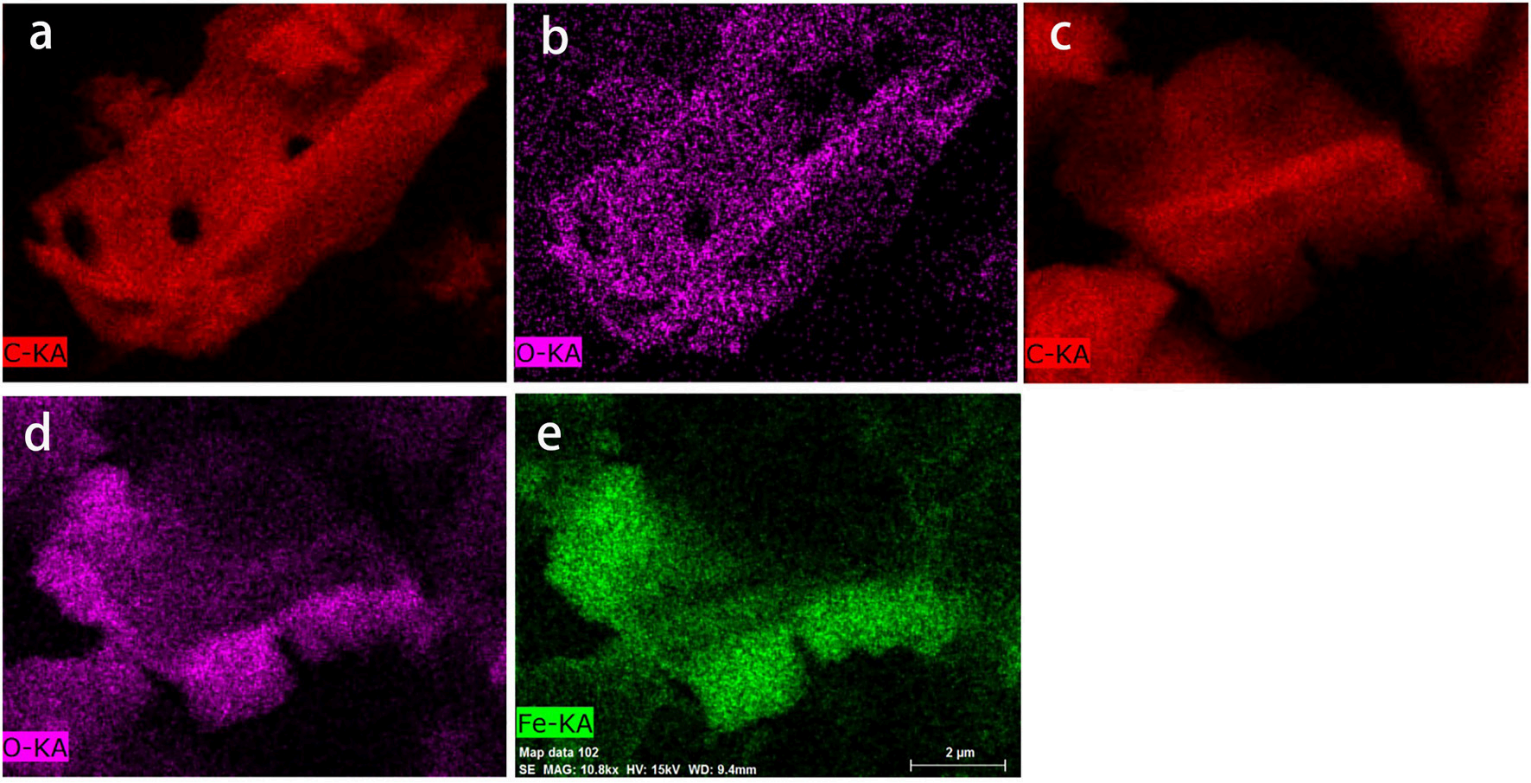
Element mapping analysis of biochar and HFO/biochar **(a)** C mapping analysis of biochar **(b)** O mapping analysis of biochar **(c)** C mapping analysis of HFO/biochar **(d)** O mapping analysis of HFO/biochar **(e)** Fe mapping analysis of HFO/biochar.

As shown in Figure 4, high temperature pyrolysis produced numerous micropores (100–300 nm) in the biochar. Comparing the SEM images of the biochar (Figure 4a) and HFO/biochar (Figure 4b), it can be seen that there were many dispersed nanoparticles in the channels of the HFO/biochar sample. EDX analysis of the biochar (Figure 4c) and HFO/biochar (Figure 4d) demonstrated that these were ferric nanoparticles, consisting of amorphous HFO. It can be seen from Figure 4c that the biochar had a high C content (91.57%), a low O content (7.05%), and traces of K. From Figure 4d it is clear that the Fe and O contents were higher (18.11 and 19.06%, respectively), and the C content lower (62.83%) for the HFO/biochar than the biochar. Fe was present as HFO loaded on the surfaces and in the channels of the biochar, increasing the O content. Element mapping (Figure 5) was carried out to determine the distribution of the different elements in the biochar and HFO/biochar, and the results indicated that Fe was homogenously dispersed in the HFO/biochar.

**Figure 6:**
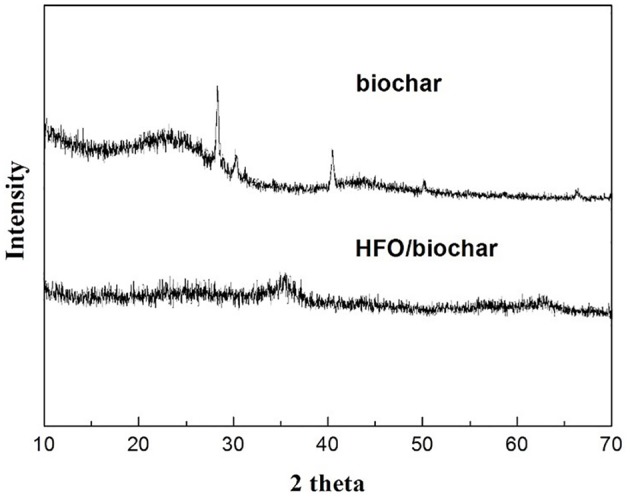
XRD analysis of biochar and HFO/biochar.

The XRD data (Figure 6) showed that the biochar and HFO/biochar were amorphous, so the HFO loaded on the surfaces of the biochar was also amorphous. Better adsorption capacities have been found for amorphous HFO on biochar surfaces than for goethite and hematite (Cumbal and Sen Gupta, 2005). Amorphous biochar, which probably has a relatively homogeneous structure, would be expected to prevent the aggregation of the HFO nanoparticles and therefore maximize the adsorption capacity of the HFO (Ying et al., 2015).

**Figure 7:**
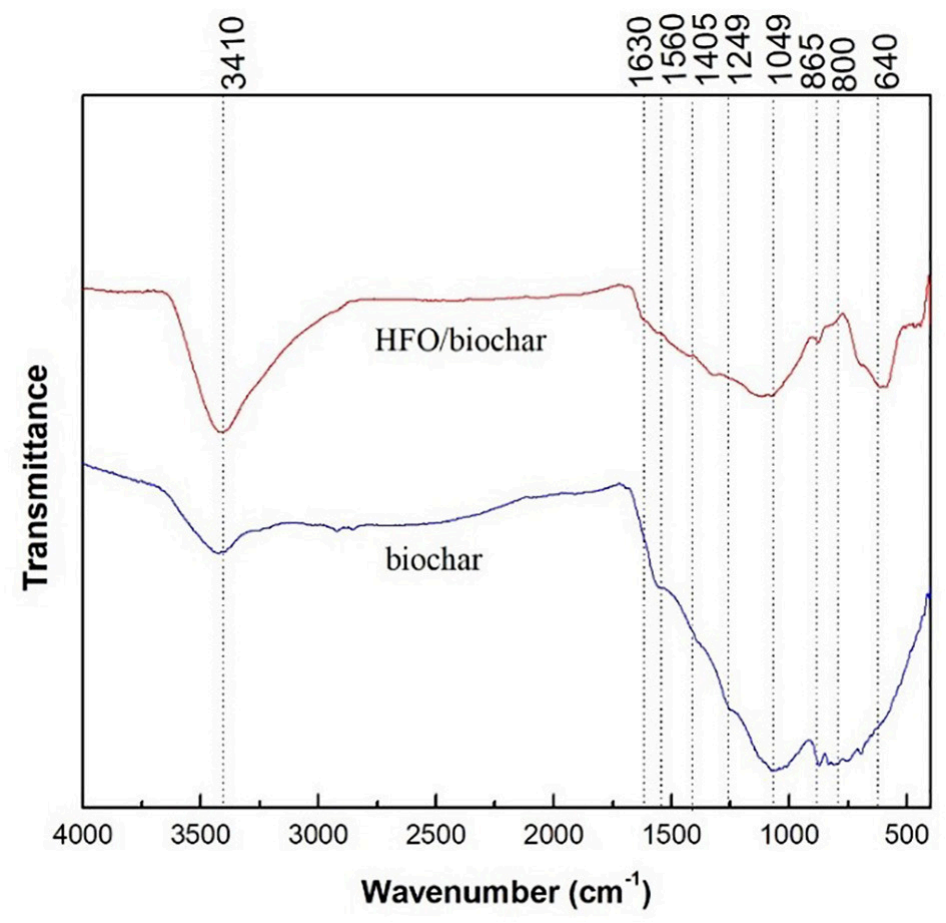
FTIR analysis of biochar and HFO/biochar.

The FTIR spectra for the biochar and HFO/biochar are shown in Figure 7. The biochar had peaks in the range 1,630–800 cm^−1^ related to C = O (1,630 cm^−1^), -COO- (1,560 cm^−1^), aromatic CO- and C-OH (1,249 cm^−1^), C-O-C and C-O in alcohols (1,049 cm^−1^), and C-Cl (800 cm^−1^) (Chen et al., 2011; Fang et al., 2014). Impregnation with HFO caused few changes to the spectrum. However, the peak at approximately 3410 cm^−1^ became more intense, indicating the concentration of hydroxyl groups on the HFO/biochar surfaces increased, demonstrating that the biochar had been successfully modified with HFO.

**Figure 8:**
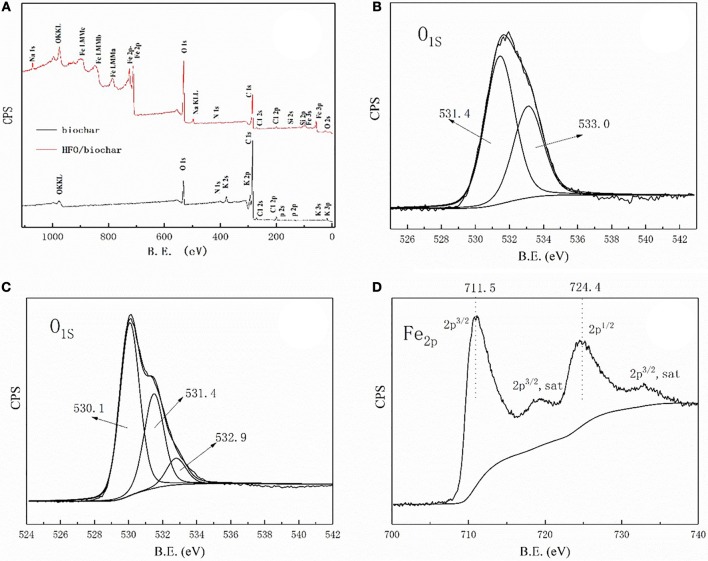
X-ray photoelectron spectroscopy analysis of biochar, HFO/biochar **(A)** XPS wide scan spectra of biochar and HFO/biochar **(B)** O1s core-level spectra of biochar **(C)** O1s core-level spectra of HFO/biochar **(D)** Fe2p core-level spectra of HFO/biochar.

The XPS spectra of the biochar and HFO/biochar were used to determine whether various elements were present (Figure 8). As can be seen from Figure 8A, both materials were scanned over a wide spectral range. The biochar surfaces mainly consisted of C and O, whereas the HFO/biochar was loaded with Fe and had an O content of 31.28% (the O content was 12.15% before HFO had been added) (Table S3). The O 1s XPS spectrum for the biochar indicated there were two forms of C with peaks at 533.0 and 531.4 eV (Figure 8B) attributed to C-O-C (Bhatia et al., 1988) and C = O, respectively (Matienzo, 1991). The HFO/biochar O 1s XPS spectrum was markedly different (Figure 8C). The 531.4 eV peak was more intense and there was a new peak at around 531.2 eV. O and iron oxyhydroxide peaks have been found at 530.1 and 531.2 eV. The Fe 2p XPS spectrum for the HFO/biochar contained peaks at 711.5 and 724.4 eV that were attributed to Fe in iron oxyhydroxide (Figure 8D). Elemental analysis data for these materials suggested that Fe was loaded onto the biochar surfaces as iron oxyhydroxide.

#### Adsorption kinetics

**Figure 9:**
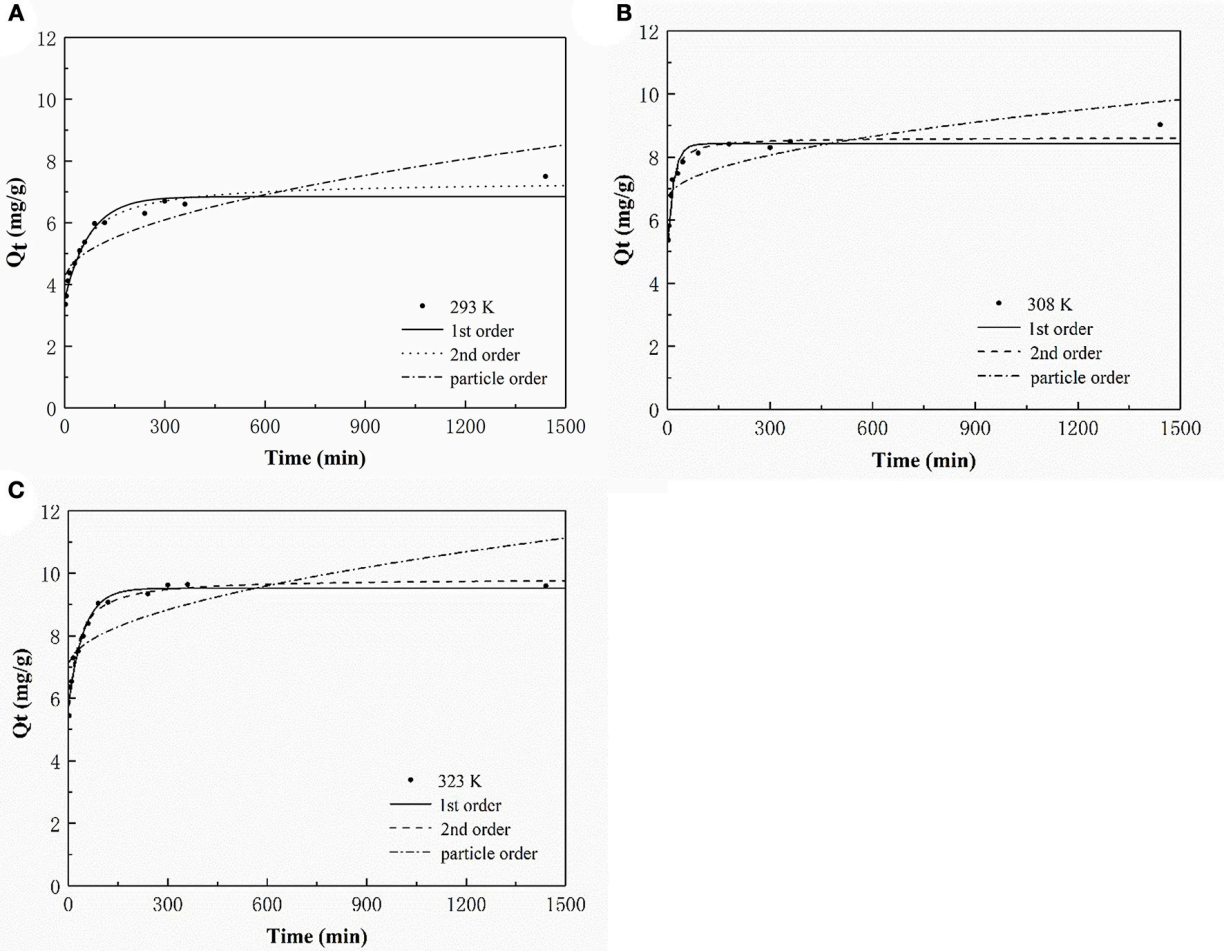
P adsorption kinetic analysis of HFO/biochar **(A)** 293K **(B)** 308K **(C)** 323K.

Three models were fitted to the P adsorption kinetics of the HFO/biochar (Figure 9). The process was confirmed to follow pseudo second order kinetics [Equation (2)], indicating that P adsorption was primarily controlled by chemical processes (Fang et al., 2015). The kinetic parameters calculated using the three kinetic models for P adsorption by the HFO/biochar are summarized in Table 2.

**Table 2:** P adsorption kinetic parameters of HFO/biochar.

**Temperature**	**1st-order**			**2nd-order**			**Particle-order**		
	**Qe**	**k_1_**	***R*^2^**	**Qe**	**K_2_**	***R*^2^**	**K_id_**	**C**	***R*^2^**
20	6.84	0.0133	0.945	7.06	0.00234	0.978	0.113	4.12	0.757
35	8.42	0.0553	0.917	8.62	0.0143	0.965	0.082	6.64	0.551
50	9.52	0.0619	0.964	9.83	0.0401	0.973	0.107	6.98	0.531

The linear expression of the pseudo first order kinetics equation is.

(3) $$\begin{array}{l} \ln\left(Q_{e}-Q_{t}\right)=\ln\;Q_{e}-k_{1}t \end{array}$$

The linear expression of the pseudo second order kinetics equation is.

(4) $$\begin{array}{l} \frac{t}{Q_{t}}=\frac{1}{k_{2}{Q_{e}}^{2}}+\frac{t}{Q_{e}} \end{array}$$

The particle diffusion equation is.

(5) $$\begin{array}{l} Q_{t}=k_{id}t^{\frac{1}{2}}+C \end{array}$$

In these equations, *Q*_e_ (mg/g) is the amount of P adsorbed at the adsorption equilibrium, *Q*_t_ (mg/g) is the amount of P adsorbed at time *t, k*_1_ (h^−1^), *k*_2_ [g/(mg·h)], and *k*_*id*_ [g/(mg·h)] are the reaction rate constants, and *C* is a constant related to the boundary layer thickness.

During the first 90 min of adsorption, the amount of P adsorbed by the HFO/biochar increased markedly over time. The initial sorption rate was faster for the HFO/biochar than for the biochar, suggesting that physisorption played the most important role in phosphate recovery (Mosa et al., 2018). HFO nanoparticles are highly selective toward anionic contaminants such as phosphate (Pan et al., 2009; Zhang et al., 2018). Negatively charged P species (usually H_2_${\text{PO}}_{4}^{-}$ or ${\text{HPO}}_{4}^{2-}$) will therefore be selectively trapped by HFO through the formation of inner-sphere complexes, and ${\text{HPO}}_{4}^{2-}$ will tend to form stronger bidentate complexes than H_2_${\text{PO}}_{4}^{-}$. In fact, all anions, including H_2_${\text{PO}}_{4}^{-}$, ${\text{HPO}}_{4}^{2-}$ and ${\text{PO}}_{4}^{3-}$, will effectively adsorb to the biochar through electrostatic interactions or through the formation of outer-sphere complexes. HFO interacts with H_2_${\text{PO}}_{4}^{-}$, ${\text{HPO}}_{4}^{2-}$, or ${\text{PO}}_{4}^{3-}$ mainly through the formation of inner-sphere complexes within the coordination sphere of Fe(III). Moreover, HFO increases the number of hydroxyl groups on the biochar surfaces, encouraging reactions with P and therefore promoting the adsorption of P (He et al., 2017). A shortage of electrostatic charges on the biochar surfaces will mean that the adsorption of P will occur primarily via the cellular structure and hydrogen-bonding interactions. The amount of P adsorbed increased slightly after 90 min and reached equilibrium after 300 min. A decrease in available adsorption sites on the HFO/biochar hindered further adsorption of P. The particle-diffusion fitting equations for the HFO/biochar did not pass through the origin, indicating that P adsorption by the HFO/biochar was influenced by internal particle diffusion.

#### Adsorption isotherms

**Figure 10:**
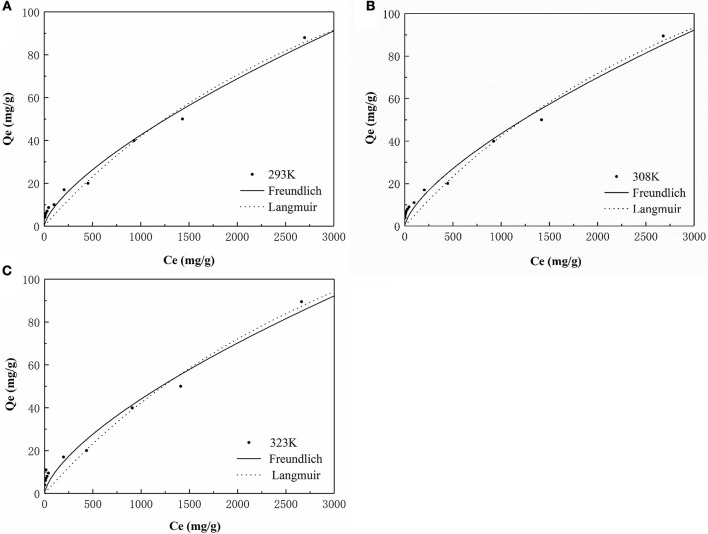
P adsorption isotherm analysis of HFO/biochar **(A)** 293K **(B)** 308K **(C)** 323K.

The isotherm for the adsorption of P onto the HFO/biochar was described using isotherm equations. The results of these analyses (Figure 10) indicated that the Freundlich model best described the experimental data, and the maximum amounts of P adsorbed onto the HFO/biochar were 225.08, 235.44, and 242.21 mg/g at 293, 308, and 323 K, respectively. It exhibits great adsorption capacity when compared with other modified biochar adsorbents. Zhu et al. (2016) studied on the characteristics of bismuth impregnated biochar and found the adsorption capacity of P was 125.40 mg/g.

The Freundlich adsorption isotherm describes non-ideal adsorption on a non-uniform surface and can be expressed as

(6) $$\begin{array}{l} Q_{e}=K_{F}{C_{e}}^{1/n} \end{array}$$

The Langmuir adsorption isotherm describes monolayer adsorption onto a uniform surface and is written

(7) $$\begin{array}{l} Q_{\text{e}}=\frac{Q_{\text{m}}K_{\text{L}}C_{\text{e}}}{1+K_{\text{L}}C_{\text{e}}} \end{array}$$

In these equations *K*_F_ [(L/g)^1/n^] and *K*_L_ (L/g) are constants for the Freundlich and Langmuir adsorption isotherm equations, respectively, 1/*n* is a parameter related to the strength of the reaction between the adsorbed species and the adsorbent surface, and *Q*_m_ (mg/g) is the monolayer adsorption capacity.

As shown in Table 3, the two isotherm models fitted the data well (*R*^2^>0.9), indicating that the adsorption of P onto the HFO/biochar was controlled by multiple steps. K_F_ (the Freundlich equation constant) increased as the temperature increased, reflecting the ability of the HFO/biochar to adsorb more P as the temperature increased. The reaction between hydroxyl ions on the HFO/biochar surfaces and negatively charged P species such as H_2_${\text{PO}}_{4}^{-}$, ${\text{HPO}}_{4}^{2-}$, and ${\text{PO}}_{4}^{3-}$ became stronger during the P adsorption process. Increasing the temperature will increase the mass transfer rate for P in the liquid phase and expand the outer layer of the HFO/biochar particles, making it easier for P to access the internal pore canals of the HFO/biochar. The Freundlich adsorption intensity parameter 1/*n* is an empirical parameter representing the heterogeneity of the site energies. 1/*n* is the Freundlich adsorption intensity parameter. 1/*n* could be used to represent heterogeneity in the site energies and in the adsorption intensity, and could be divided into five levels (Tseng and Wu, 2008). A smaller 1/n value will indicate stronger adsorptive ability, so the ability of the HFO/biochar to adsorb P increased as the temperature increased. The 1/n values for the Freundlich models fitted to the experimental data over the range 0.5–1 indicated that adsorption of P onto the HFO/biochar was favorable. This indicated that the HFO/biochar could adsorb large amounts of P even at low P concentrations. It is possible that electrostatic attraction would make a marginal contribution to the sorption of P because the HFO/biochar was less electronegative than the biochar (Mosa et al., 2018).

**Table 3:** P adsorption isotherm parameters of HFO/biochar.

**Temperature**	**Freundlich**			**Langmuir**		
	**K_F_**	**1/n**	***R*^2^**	**K_L_**	**Q_m_**	***R*^2^**
293	0.354	0.693	0.981	0.229	225.08	0.981
308	0.386	0.684	0.974	0.220	235.44	0.951
323	0.451	0.664	0.962	0.212	242.21	0.939

#### Thermodynamic calculations

The thermodynamics of the adsorption of P onto the HFO/biochar at 293, 308, and 323 K were analyzed. The Freundlich model was used to calculate the differential adsorption enthalpy (ΔH), adsorption free energy (ΔG), and adsorption entropy (ΔS).

The adsorption enthalpy is closely related to the amount adsorbed. Using an initial amount adsorbed of 1, the adsorption enthalpy will be ΔH. The formula can be expressed as (Rouquerol et al., 2013)

(8) $$\begin{array}{l} \ln\left(\frac{1}{C_{\text{e}}}\right)=\ln\;K^{\prime }-\frac{\Delta H}{RT} \end{array}$$

were *R* [8.314 J/(mol·K)] is the ideal gas constant, *T* (K) is the thermodynamic temperature, and *K*′ is a constant.

Δ*G* can be calculated using the Gibbs equation, which can be expressed as

(9) $$\Delta G=-RT\int_{0}^{x}\left(\frac{q^{\prime }}{x}\right)dx$$

were *x* is the mole fraction of the solute in the solution and *q*′ (mmol/g) is the amount adsorbed. Given that Δ*G* is not related to *q*′ the formula can be expressed as

(10) $$\begin{array}{l} \Delta G=-nRT \end{array}$$

Δ*S* can be calculated from Δ*H* and Δ*G* using the equation

(11) $$\begin{array}{l} \Delta S=\frac{\left(\Delta H-\Delta G\right)}{T} \end{array}$$

The calculated ΔH, ΔG, and ΔS values for the HFO/biochar are shown in Table 4. ΔH was positive, indicating that the adsorption process was endothermic. This confirmed that increasing the temperature would increase the amount adsorbed and increase the rate at which P was adsorbed onto HFO/biochar. ΔG was < zero, indicating that P would spontaneously migrate from the solution to the HFO/biochar surfaces. According to Table 4, ΔG decreased as the temperature increased, indicating that the P adsorption efficiency would increase as the temperature increased (Chen and Lu, 2014). ΔS for the HFO/biochar was positive, indicating that disorder at the solid-liquid interface increased during adsorption and that P would tend to adsorb onto the HFO/biochar surfaces. This could probably be attributed to the reaction between P and active adsorption sites on the HFO particles (Ünlü and Ersoz, 2006). Adsorption and desorption commonly occur simultaneously. ΔS will depend on both adsorption and desorption, increasing during adsorption processes and decreasing during desorption processes. The overall decrease that was observed therefore meant that the decrease in ΔS associated with adsorption was much greater than the increase in ΔS associated with desorption at higher temperatures. This conclusion was consistent with the results of a previous study (He et al., 2017).

**Table 4:** P adsorption thermodynamics calculation parameters of HFO/biochar.

**T (K)**	**ΔH (KJ/mol)**	**ΔG (kJ/mol)**	**ΔS (J/(mol·K))**
293	0.202	−21.602	80.630
308		−22.605	79.960
323		−23.606	79.347

### Estimation of the fate of phosphorus

Recovering P from swine manure is very important. In this section, calculations are performed to analyze P solubilization and release from swine manure and the capture of P through struvite crystallization and adsorption to HFO/biochar. The calculated P mass balances for the swine manure treatment process are shown in Table 5. The proportion of P recovered from the manure was estimated from the P flow during solubilization and capture.

**Table 5:** P mass balance calculation.

**Treatment**		**P solubilization**			**P capture**			
		**TP (mg/L)**	**IP (mg/L)**	**IP/TP (%)**	**IP (mg/L)**			**IP/TP (%)**
					**Struvite**	**HFO/biochar**	**Total**	
H_2_O_2_		230.3	48.0	20.8	40.8	6.5	47.3	20.5
Ultrasound			55.0	23.9	46.8	7.4	54.2	23.5
Ultrasound/H_2_O_2_	pH 7.5		87.0	37.8	74.0	11.7	85.7	37.2
	pH 5.0		185.2	80.4	157.4	25.0	182.4	79.2
	pH 3.0		211.3	91.7	179.6	28.5	208.1	90.4

During the solubilization of P during ultrasound/H_2_O_2_ digestion, OP and sparingly soluble P will be decomposed, dissolved, and released. The results showed that the best P solubilization performance was achieved during performing the ultrasound/H_2_O_2_ process at pH 3. The amount of IP released into the supernatant reached 211.3 mg/L, corresponding to 91.7% of the TP. With regard to the capture of P, the struvite crystallization ratio and HFO/biochar adsorption ratio were assumed to be 85.0 and 90.0%, respectively, when the P flow was analyzed. The maximum amount of P captured by struvite crystallization was 179.6 mg/L, and the maximum amount adsorbed by the HFO/biochar was 28.5 mg/L. The maximum total amount captured (through struvite crystallization and HFO/biochar adsorption) was therefore 208.1 mg/L, corresponding to 90.4% of the TP originally in the swine manure.

## Conclusions

Ultrasound/H_2_O_2_ digestion coupled with struvite crystallization and HFO/biochar adsorption was used to recover P from swine manure. Ultrasound/H_2_O_2_ digestion efficiently solubilized both OP and sparingly soluble P species. The optimal ultrasound/H_2_O_2_ digestion efficiency was obtained at pH 3, using 5% H_2_O_2_ and an ultrasonication time of 1.5 h, and this gave an IP:TP ratio of 91.7%. The crystallization of struvite recovered 85% of the available P at pH 10.0, a Mg:P molar ratio of 1.4, and a stirring rate of 150 rpm. P adsorption onto the HFO/biochar followed pseudo second order kinetics, indicating that the adsorption of P was mainly controlled by chemical processes. The Freundlich model best explained the experimental results. The maximum amounts of P adsorbed were in the range 225.08–242.21 mg/g, and adsorption was found to be endothermic, spontaneous, and associated with increased disorder. A total of 90.4% of the P was recovered as struvite and P saturated HFO/biochar. The present work has major implications for cleaner production. It not only achieves environmentally friendly disposal of swine manure but also recovers the P resource. The recover struvite and adsorbed HFO/biochar yield a nutrient-rich product as a potential fertilizer for soil application and agriculture production. Moreover, the use of corn waste to produce HFO/biochar reduces agricultural waste disposal and minimizes CO_2_ emission.

## Author contributions

TZ and QW finished the experiments, analyzed the data, and wrote the manuscript. TZ and YD proposed the idea, conceived and conducted the research, analyzed the results, modified and refinement the manuscript. RJ proposed the idea, designed the study, and modified the manuscript. All authors reviewed the manuscript.

### Conflict of interest statement

The authors declare that the research was conducted in the absence of any commercial or financial relationships that could be construed as a potential conflict of interest.

## References

[B1] APHA (2012). Standard methods for the examination of water and wastewater. 22nd Edn. Washington, DC: Water Environment Federation.

[B2] Appels L. Baeyens J. Degreve J. Dewil R. (2008). Principles and potential of the anaerobic digestion of waste-activated sludge. Prog. Energy Combust. Sci. 34, 755–781. 10.1016/j.pecs.2008.06.002

[B3] Bhatia Q. S. Pan D. H. Koberstein J. T. (1988). Preferential surface adsorption in miscible blends of polystyrene and poly (vinyl methyl ether). Macromolecules 21, 2166–2175. 10.1021/ma00185a049

[B4] Chen W. Westerhoff P. Leenheer J. A. Bookash K. (2003). Fluorescence excitation-emission matrix regional integration to quantify spectra for dissolved organic matter. Environ. Sci. Technol. 37, 5701–5710. 10.1021/es034354c 14717183

[B5] Chen X. Chen G. Chen L. Chen Y. Lehmann J. Mcbride M. B.. (2011). Adsorption of copper and zinc by biochars produced from pyrolysis of hardwood and corn straw in aqueous solution. Bioresour. Technol. 102, 8877–8884. 10.1016/j.biortech.2011.06.078 21764299

[B6] Chen Y. C. Lu C. (2014). Kinetics, thermodynamics and regeneration of molybdenum adsorption in aqueous solutions with NaOCl-oxidized multiwalled carbon nanotubes. J. Ind. Eng. Chem. 20, 2521–2527. 10.1016/j.jiec.2013.10.035

[B7] Cumbal L. Sen Gupta A. K. (2005). Arsenic removal using polymer-supported hydrated iron (III) oxide nanoparticles: role of Donnan membrane effect. Environ. Sci. Technol. 39, 6508–6515. 10.1021/es050175e 16190206

[B8] Dai L. Tan F. Wu B. He M. Wang W. Tang X.. (2015). Immobilization of phosphorus in cow manure during hydrothermal carbonization. J. Environ. Manage. 157, 49–53. 10.1016/j.jenvman.2015.04.009 25881151

[B9] Dargahi A. Pirsaheb M. Hazrati S. Fazlzadehdavil M. Khamutian R. Amirian T. (2015). Evaluating efficiency of H2O2 on removal of organic matter from drinking water. Desal. Water Treat. 54, 1589–1593. 10.1080/19443994.2014.889608

[B10] Ekpo U. Ross A. B. Camargo-Valero M. A. Fletcher L. A. (2016a). Influence of pH on hydrothermal treatment of swine manure: impact on extraction of nitrogen and phosphorus in process water. Bioresour. Technol. 214, 637–644. 10.1016/j.biortech.2016.05.012 27187568

[B11] Ekpo U. Ross A. B. Camargovalero M. A. Williams P. T. (2016b). A comparison of product yields and inorganic content in process streams following thermal hydrolysis and hydrothermal processing of microalgae, manure and digestate. Bioresour. Technol. 200, 951–960. 10.1016/j.biortech.2015.11.018 26615335

[B12] Elser J. Bennett E. (2011). Phosphorus cycle: a broken biogeochemical cycle. Nature. 478, 29–31. 10.1038/478029a 21979027

[B13] Fang C. Zhang T. Li P. Jiang R. Wu S. B. Nie H. Y.. (2015). Phosphorus recovery from biogas fermentation liquid by Ca-Mg loaded biochar. J. Environ. Sci. 29, 106–114. 10.1016/j.jes.2014.08.019 25766018

[B14] Fang C. Zhang T. Li P. Jiang R. F. Wang Y. C. (2014). Application of magnesium modified corn biochar for phosphorus removal and recovery from swine wastewater. Inter. J. Environ. Res. Pub. Heal. 11, 9217–9237. 10.3390/ijerph110909217 25198685 PMC4199016

[B15] Gifford M. K. Liu J. Rittmann B. E. Vannela R. Westerhoff P. (2015). Phosphorus recovery from microbial biofuel residual using microwave peroxide digestion and anion exchange. Water Res. 70, 130–137. 10.1016/j.watres.2014.11.052 25528543

[B16] Gogate P. R. Pandit A. B. (2004). A review of imperative technologies for wastewater treatment II: hybrid methods. Adv. Environ. Res. 8, 553–597. 10.1016/S1093-0191(03)00031-5

[B17] Gong C. Jiang J. Li D. (2015). Ultrasound coupled with Fenton oxidation pre-treatment of sludge to release organic carbon, nitrogen and phosphorus. Sci. Total Environ. 532, 495–500. 10.1016/j.scitotenv.2015.05.131 26100728

[B18] He X. Zhang T. Ren H. Li G. Ding L. Pawlowski L. (2017). Phosphorus recovery from biogas slurry by ultrasound/H2O2 digestion coupled with HFO/biochar adsorption process. Waste Manage. 60, 219–229. 10.1016/j.wasman.2016.08.032 27594573

[B19] Jiang J. Gong C. Tian S. Yang S. Zhang Y. (2014a). Impact of ultrasonic treatment on dewaterability of sludge during Fenton oxidation. Environ. Monit. Assess. 186, 8081–8088. 10.1007/s10661-014-3988-y 25108663

[B20] Jiang J. Gong C. Wang J. Tian S. Zhang Y. (2014b). Effects of ultrasound pre-treatment on the amount of dissolved organic matter extracted from food waste. Bioresour. Technol. 155, 266–271. 10.1016/j.biortech.2013.12.064 24457300

[B21] Jin Y. Hu Z. Wen Z. (2009). Enhancing anaerobic digestibility and phosphorus recovery of dairy manure through microwave-based thermochemical pretreatment. Water Res. 43, 3493–3502. 10.1016/j.watres.2009.05.017 19555991

[B22] Jiraroj D. Unob F. Hagege A. (2006). Degradation of Pb–EDTA complex by a H2O2/UV process. Water Res. 40, 107–112. 10.1016/j.watres.2005.10.041 16364402

[B23] Khanal S. K. Grewell D. Sung S. Van L. J. (2007). Ultrasound applications in wastewater sludge pretreatment: a review. Crit. Rev. Environ. Sci. Technol. 37, 277–313. 10.1080/10643380600860249

[B24] Kim M. Kim D. (2016). Selective release of phosphorus from waste-activated sludge by low-temperature thermal treatment: comparative study with ultrasonic treatment. Desalin. Water Treat. 57, 24109–24115. 10.1080/19443994.2016.1145954

[B25] Kobayashi Y. Hayashi M. Yoshino F. Tamura M. Yoshida A. Ibi H.. (2014). Passive ultrasonic irrigation in the presence of a low concentration of hydrogen peroxide enhances hydroxyl radical generation and bactericidal effect against *Enterococcus faecalis*. J. Oral Sci. 56, 35–39. 10.2334/josnusd.56.35 24739706

[B26] Liu P. Li C. Liang X. Xu J. Lu G. Ji F. (2013). Advanced oxidation of hypophosphite and phosphite using a UV/H2O2 process. Environ. Technol. 34, 2231–2239. 10.1080/09593330.2013.765917 24350477

[B27] Lou Y. Y. Ye Z. L. Chen S. H. Wei Q. S. Zhang J. Q. Ye X. (2018). Influences of dissolved organic matters on tetracyclines transport in the process of struvite recovery from swine wastewater. Water Res. 134, 311–326. 10.1016/j.watres.2018.02.010 29438892

[B28] Malgorzata K. Sabina Z. Koszelnik P. (2018). Removal of organochlorine pesticides (OCPs) from aqueous solutions using hydrogen peroxide, ultrasonic waves, and a hybrid process. Sep. Purif. Technol. 192, 457–464. 10.1016/j.seppur.2017.10.046

[B29] Matienzo L. J. (1991). Surface reactions of poly (ether ether ketone) with He2+ ions. Polymer 32, 3057–3061. 10.1016/0032-3861(91)90209-2

[B30] Mosa A. El-Ghamry A. Tolba M. (2018). Functionalized biochar derived from heavy metal rich feedstock: phosphate recovery and reusing the exhausted biochar as an enriched soil amendment. Chemosphere 198, 351–363. 10.1016/j.chemosphere.2018.01.113 29421750

[B31] Murphy K. R. Stedmon C. A. Graeber D. Bro R. (2013). Fluorescence spectroscopy and multi-way techniques. PARAFAC. Anal. Methods. 5, 6557–6566. 10.1039/c3ay41160e

[B32] Naddeo V. Borea L. Belgiorno V. (2015). Sonochemical control of fouling formation in membrane ultrafiltration of wastewater: effect of ultrasonic frequency. J. Water Process Eng. 8, 92–97. 10.1016/j.jwpe.2014.12.005

[B33] Pan B. Pan B. Zhang W. Lv L. Zhang Q. Zheng S. (2009). Development of polymeric and polymer-based hybrid adsorbents for pollutants removal from waters. Chem. Eng. J. 151, 19–29. 10.1016/j.cej.2009.02.036

[B34] Parker D. B. Cai L. Kim K. H. Hales K. E. Spiehs M. J. Woodbury B. L.. (2012). Reducing odorous VOC emissions from swine manure using soybean peroxidase and peroxides. Bioresour. Technol. 124, 95–124. 10.1016/j.biortech.2012.08.031 22985851

[B35] Pehkonen S. O. Zhang Q. (2002). The degradation of organophosphorus pesticides in natural waters: a critical review. Crit. Rev. Environ. Sci. Tec. 32, 17–72. 10.1080/10643380290813444

[B36] Rittmann B. E. Mayer B. Westerhoff P. Edwards M. (2011). Capturing the lost phosphorus. Chemosphere 84, 846–853. 10.1016/j.chemosphere.2011.02.001 21377188

[B37] Rouquerol J. Rouquerol F. Llewellyn P. Maurin G. Sing K. S. (2013). Adsorption by Powders and Porous Solids: Principles, Methodology and Applications. Cambridge, MA: Academic Press.

[B38] Ruiz-Hernando M. Cabanillas E. Labanda J. Llorens J. (2015). Ultrasound, thermal and alkali treatments affect extracellular polymeric substances (EPSs) and improve waste activated sludge dewatering. Process Biochem. 50, 438–446. 10.1016/j.procbio.2015.01.001

[B39] Srinivasan A. Liao P. H. Lo K. V. Harada S. (2015). Optimization of radiofrequency-oxidation treatment of dairy manure. J. Environ. Chem. Eng. 3, 2155–2160. 10.1016/j.jece.2015.07.024

[B40] Tseng R. L. Wu F. C. (2008). Inferring the favorable adsorption level and the concurrent multi-stage process with the Freundlich constant. J. Hazard. Mater. 155, 277–287. 10.1016/j.jhazmat.2007.11.061 18178000

[B41] Ünlü N. Ersoz M. (2006). Adsorption characteristics of heavy metal ions onto a low cost biopolymeric sorbent from aqueous solutions. J. Hazard. Mater. 36, 272–280. 10.1016/j.jhazmat.2005.12.013 16442227

[B42] Uslu M. O. Balcioglu I. A. (2009). Comparison of the ozonation and Fenton process performances for the treatment of antibiotic containing manure. Sci. Total Environ. 407, 3450–3458. 10.1016/j.scitotenv.2009.01.045 19232678

[B43] Withers P. J. A. Elser J. J. Hilton J. Ohtake H. Schipper W. J. Van Dijk K. C. (2015). Greening the global phosphorus cycle: how green chemistry can help achieve planetary P sustainability. Green Chem. 17, 2087–2099. 10.1039/C4GC02445A

[B44] Ying B. Lin G. Jin L. Zhao Y. Zhang T. Tang J. (2015). Adsorption and degradation of 2, 4-dichlorophenoxyacetic acid in spiked soil with Fenanoparticles supported by biochar. Acta Agr Scand B-S.P. 65, 215–221. 10.1080/09064710.2014.992939

[B45] Zhang C. Chen Y. (2009). Simultaneous nitrogen and phosphorus recovery from sludge-fermentation liquid mixture and application of the fermentation liquid to enhance municipal wastewater biological nutrient removal. Environ. Sci. Technol. 43, 6164–6170. 10.1021/es9005948 19746708

[B46] Zhang K. Gao N. Deng Y. Lin T. F. Ma Y. Li L.. (2011). Degradation of bisphenol-A using ultrasonic irradiation assisted by low-concentration hydrogen peroxide. J. Environ. Sci. 23, 31–36. 10.1016/S1001-0742(10)60397-X 21476337

[B47] Zhang T. Fang C. Li P. Jiang R. F. (2014). Application of struvite process for nutrient recovery from anaerobic digesters of livestock wastewater. Environ. Prot. Eng. 40, 29–42. 10.5277/epel40303

[B48] Zhang T. Li Q. C. Ding L. L. Ren H. Q. Xu K. Wu Y. G.. (2011). Modeling assessment for ammonium nitrogen recovery from wastewater by chemical precipitation. J. Environ. Sci. 23, 881–890. 10.1016/S1001-0742(10)60485-8 22066209

[B49] Zhang T. Xu H. Y. Li H. H. He X. Y. Shi Y. J. Kruse A. (2018). Microwave digestion-assisted HFO/biochar adsorption to recover phosphorus from swine manure. Sci. Total Environ. 621, 1512–1526. 10.1016/j.scitotenv.2017.10.077 29102181

[B50] Zhu N. Yan T. Qiao J. Cao H. (2016). Adsorption of arsenic, phosphorus and chromium by bismuth impregnated biochar: Adsorption mechanism and depleted adsorbent utilization. Chemosphere 164, 32–40. 10.1016/j.chemosphere.2016.08.036 27574812

