# Supplementary material for: Recovery of Phosphorus From Swine Manure by Ultrasound/H2O2 Digestion, Struvite Crystallization, and Ferric Oxide Hydrate/Biochar Adsorption
Source: Front Chem. 2018 Oct 8;6:464. doi: 10.3389/fchem.2018.00464 (PMC6187983; doi:10.3389/fchem.2018.00464)
Supplement: Supplementary file 1 [file Data_Sheet_1.doc]

*Appendix A. Supplementary data*

**Recovery of phosphorus from swine manure by ultrasound/H_2_O_2_ digestion, struvite crystallization, and ferric oxide hydrate/biochar adsorption**

Tao Zhang ^1,2,*^, Qiming Wang ^1^, Yaxin Deng ^1,3^, Rongfeng Jiang ^1^

^1^ Beijing Key Laboratory of Farmland Soil Pollution Prevention and Remediation, Key Laboratory of Plant-Soil Interactions of Ministry of Education, Biomass Engineering Center, College of Resources and Environmental Sciences, China Agricultural University, Beijing 100193, China

^2^ Institute for Agricultural Engineering, University of Hohenheim, Garbenstrabe 9, 70599 Stuttgart, Germany

^3^ Illinois Sustainable Technology Center, University of illinois Urbana-Champaign, Illinois 61801, USA

^*^ Corresponding author e-mail: [taozhang@cau.edu.cn](mailto:taozhang@cau.edu.cn) (Tao Zhang)

a


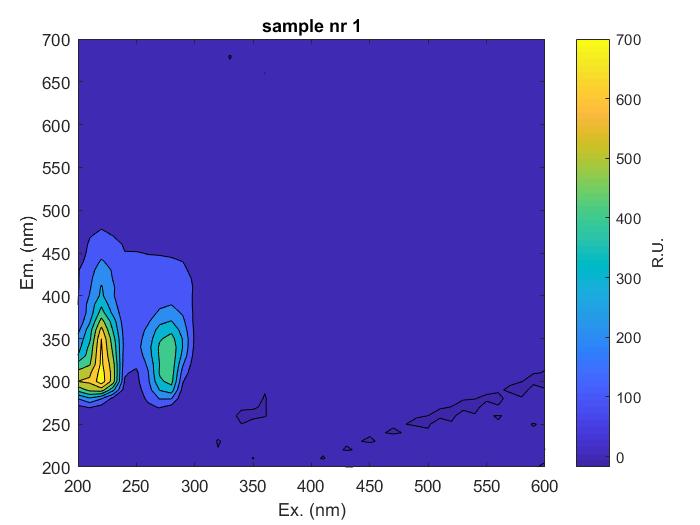


b


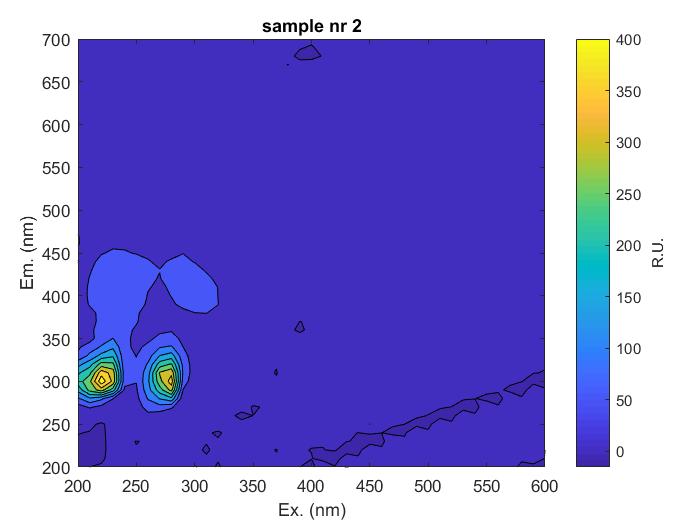


c


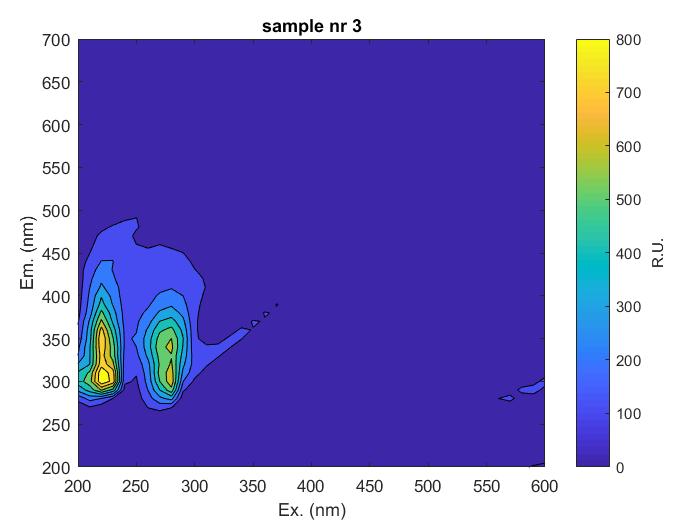


d


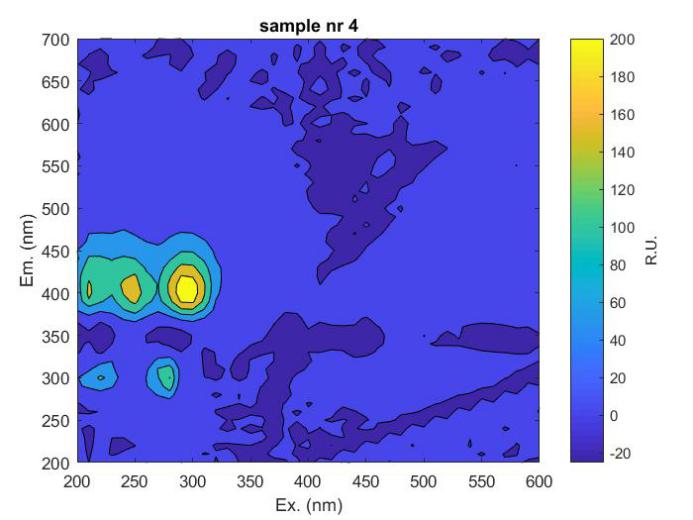


**Fig. S1** Three-dimensional excitation emission matrix analysis of swine manure supernatant at different treated processes ((a) raw swine manure, (b) swine manure treated with H_2_O_2_ (2.5 mL of 30% H_2_O_2_), (c) swine manure treated with ultrasound (303 K, 1.5 h, 100% power), (d) swine manure treated ultrasound/H_2_O_2_ digestion at pH 3.0 (303 K, 1.5 h, 100% power; 2.5 mL of 30% H_2_O_2_))

a


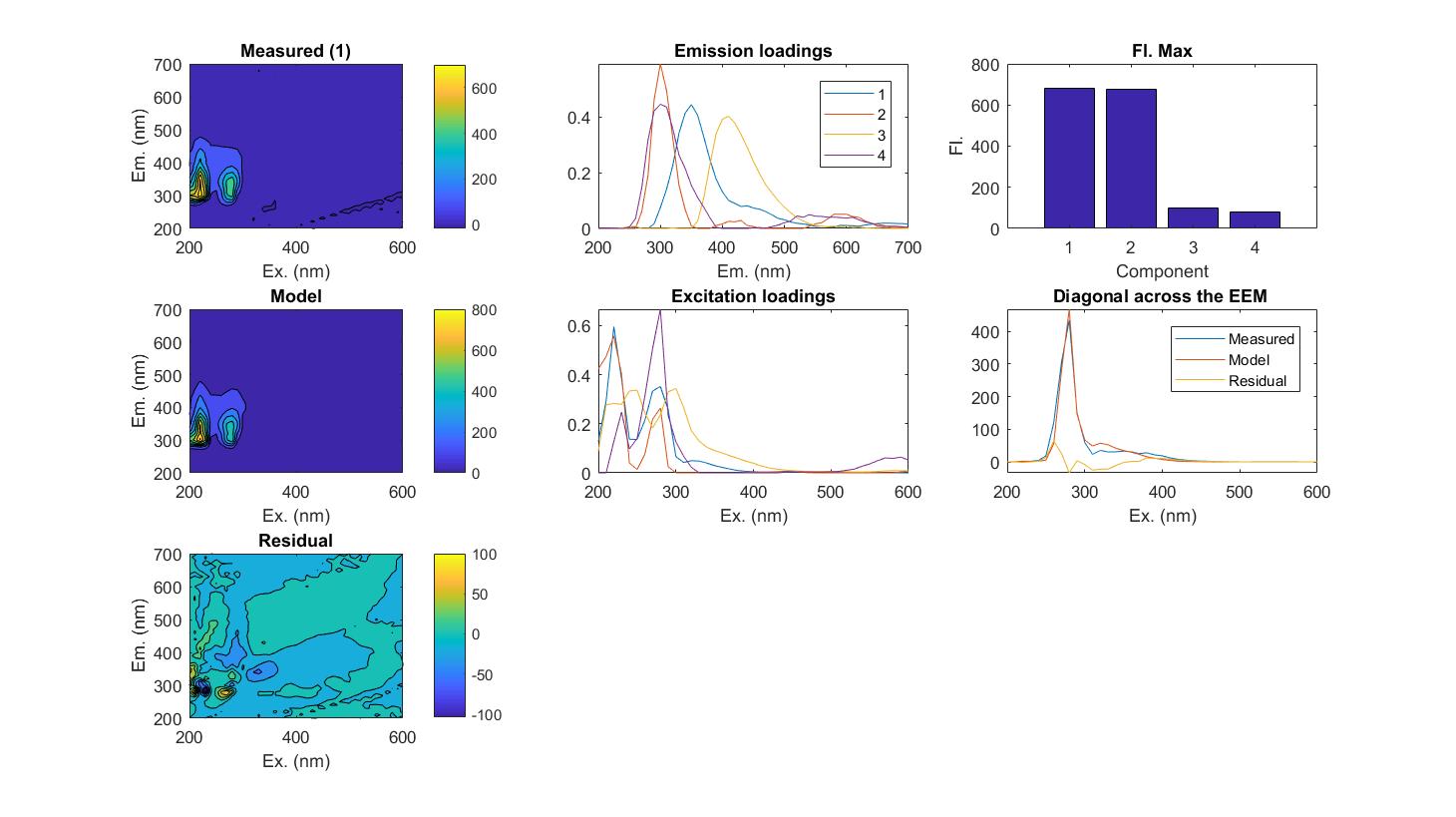


a’


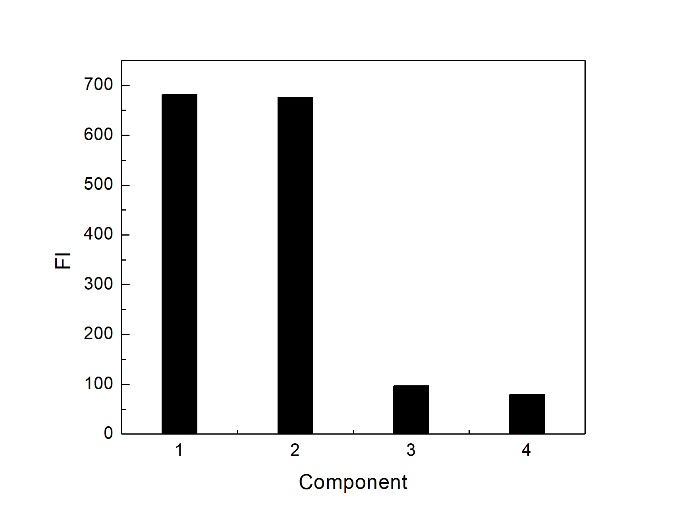


b


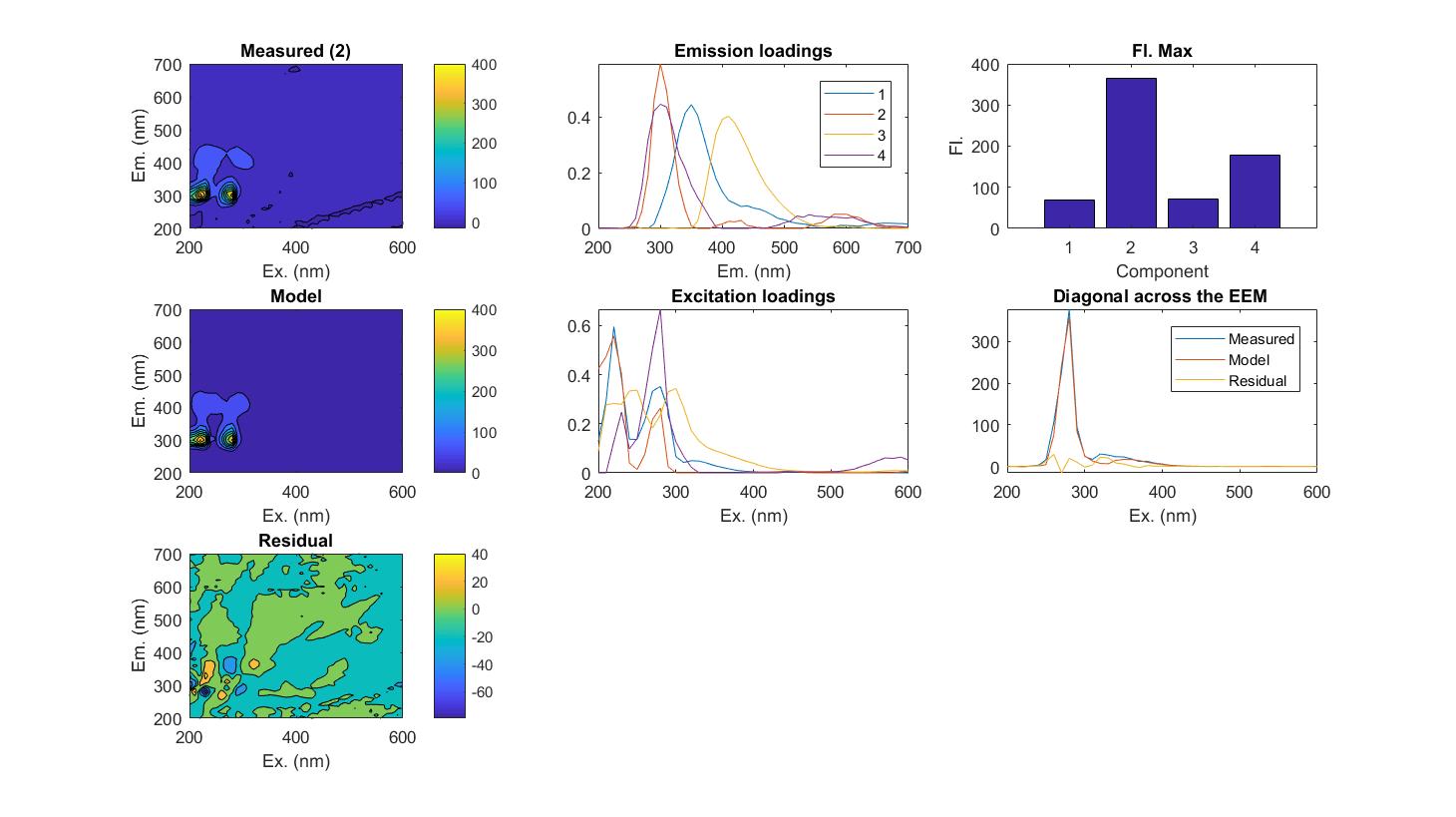


b’


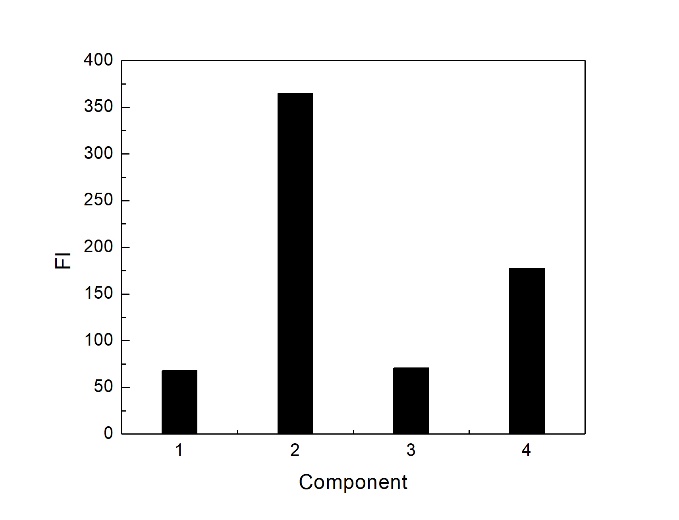


c


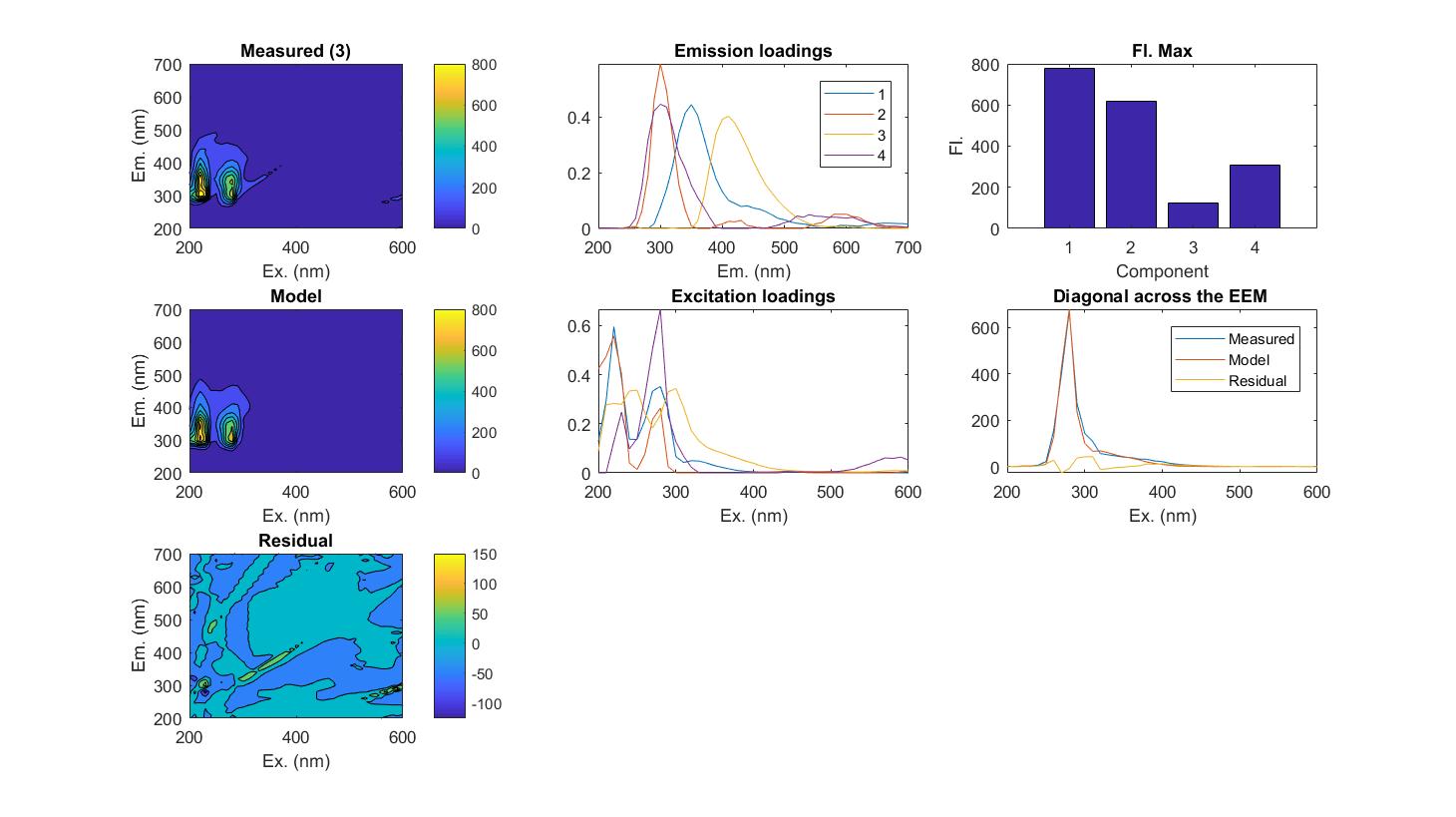


c’


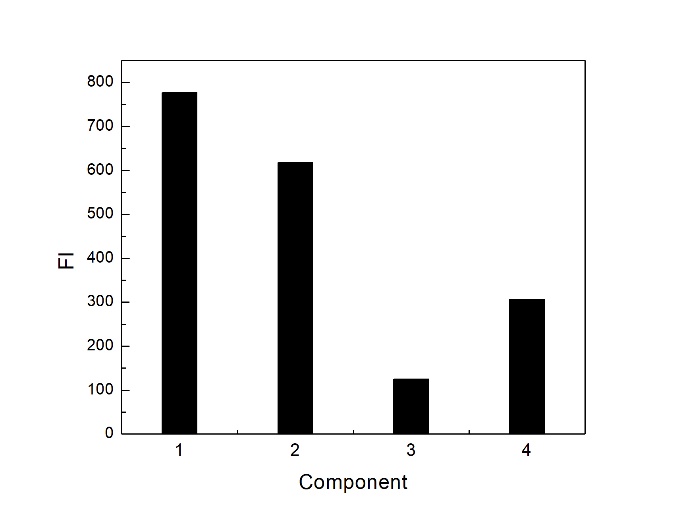


d


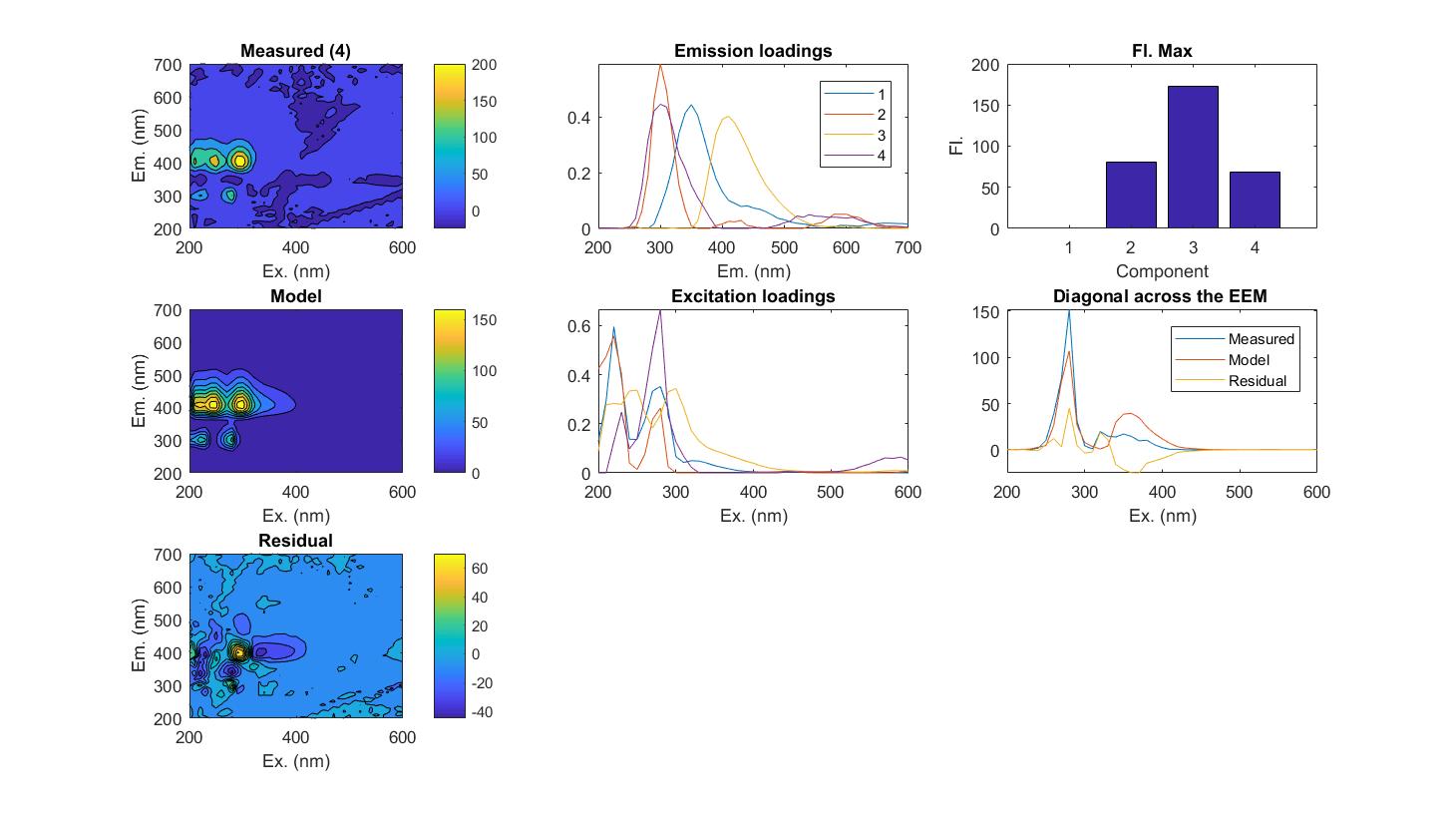


d’


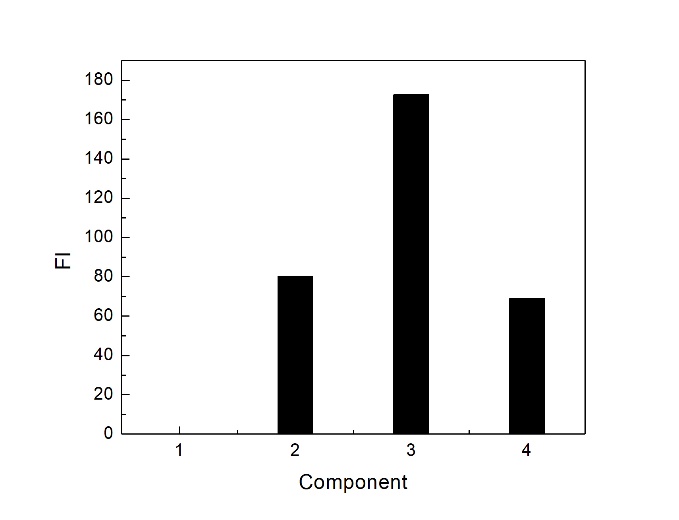


**Fig. S2** Residuals plots and fluorescence intensity in four-component model of swine manure supernatant at different treated processes ((a) raw swine manure, (b) swine manure treated with H_2_O_2_ (2.5 mL of 30% H_2_O_2_), (c) swine manure treated with ultrasound (303 K, 1.5 h, 100% power), (d) swine manure treated ultrasound/H_2_O_2_ digestion at pH 3.0 (303 K, 1.5 h, 100% power; 2.5 mL of 30% H_2_O_2_))

a


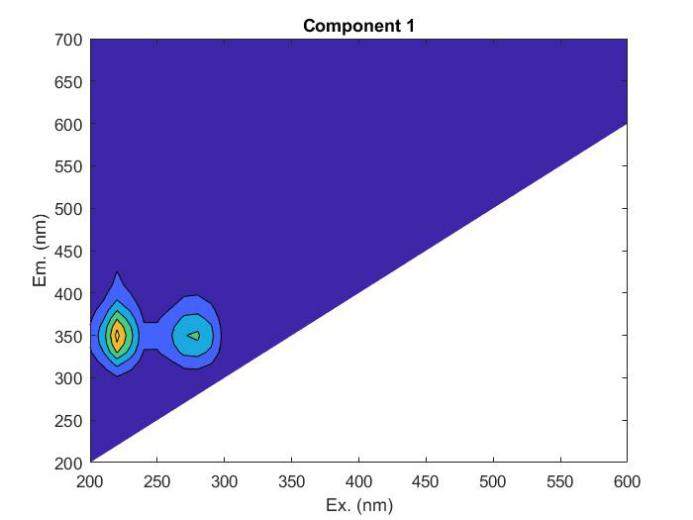


a’


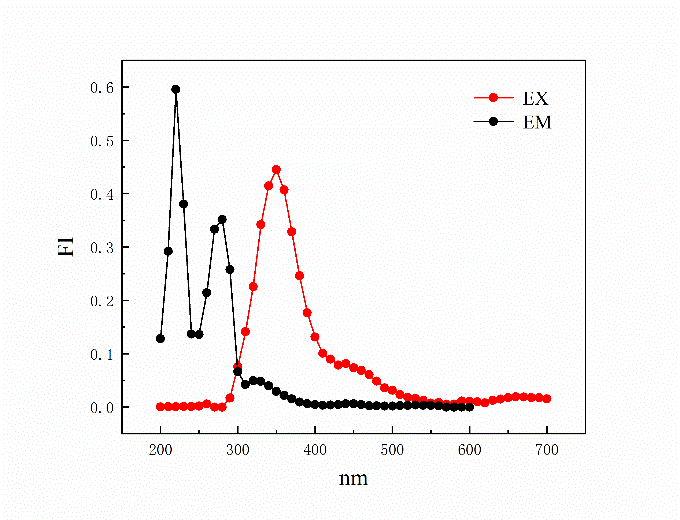


b


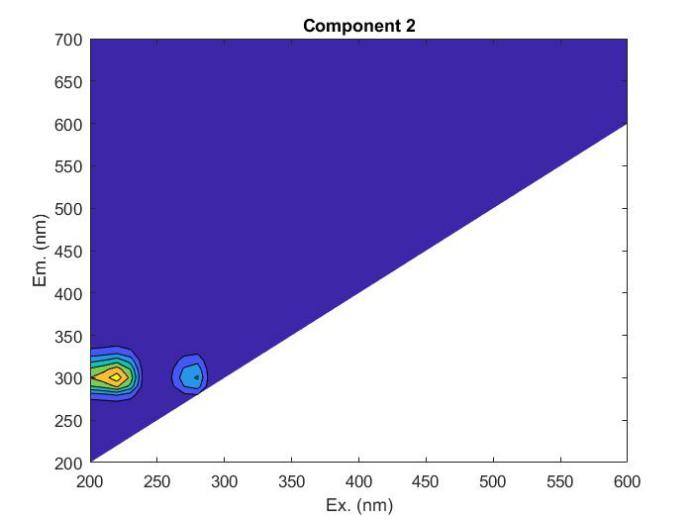


b’


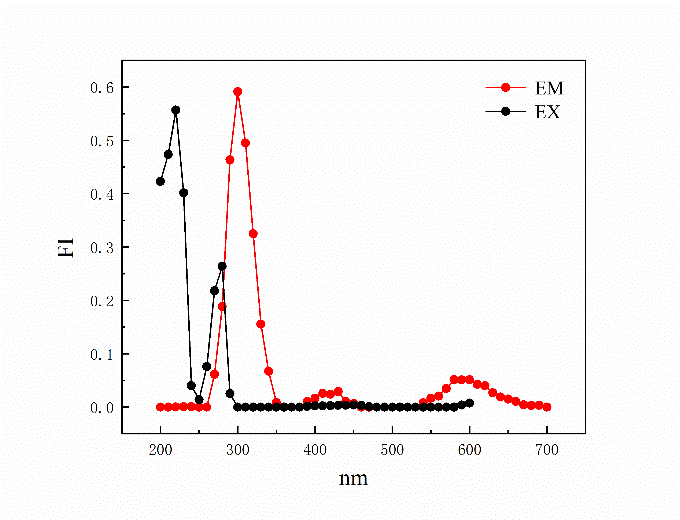


c


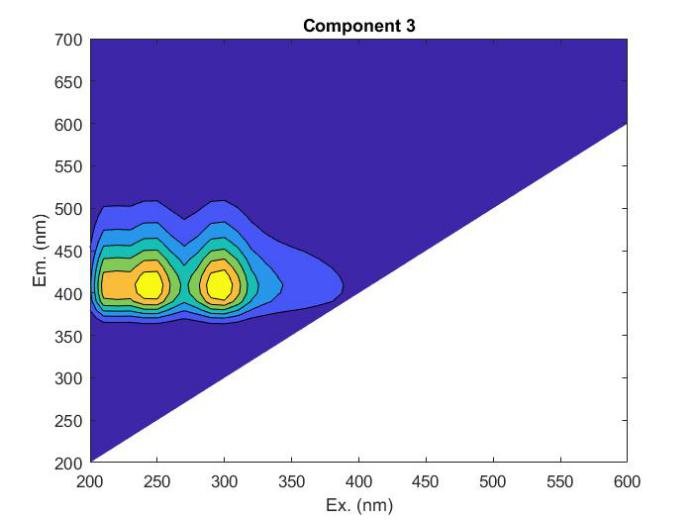


c’


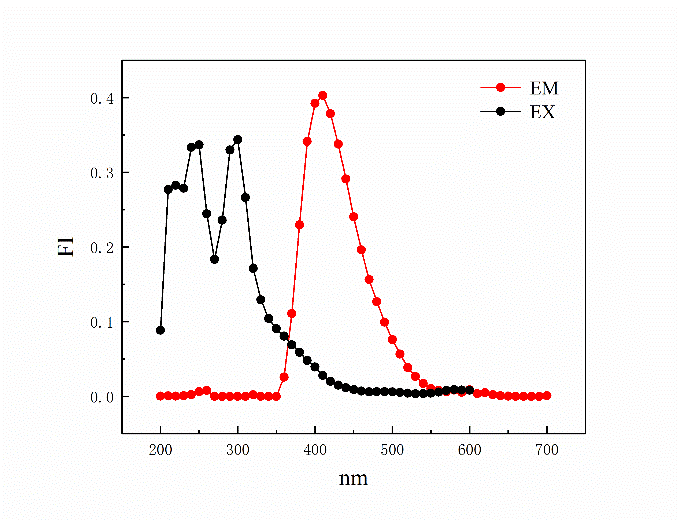


d


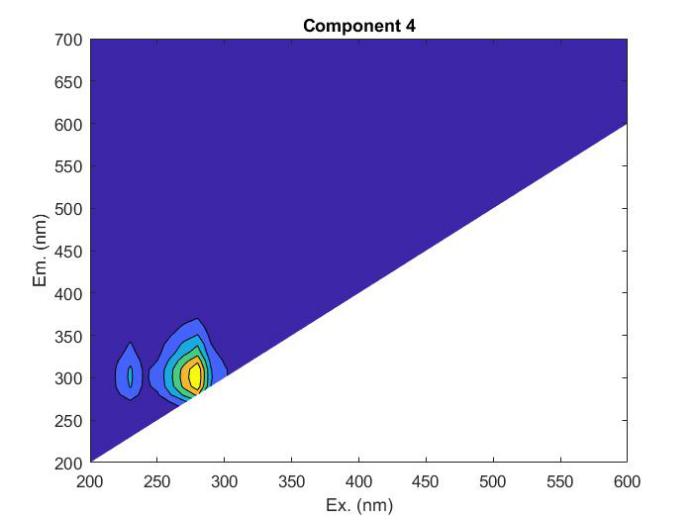


d’


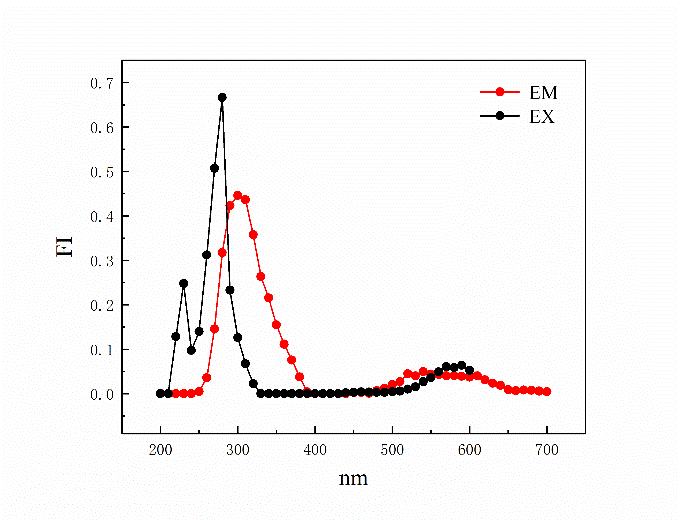


**Fig. S3** Excitation and emission spectral loadings of the four-component PARAFAC model of swine manure supernatant (a) Component 1 (b) Component 2 (c) Component 3 (d) Component 4

a


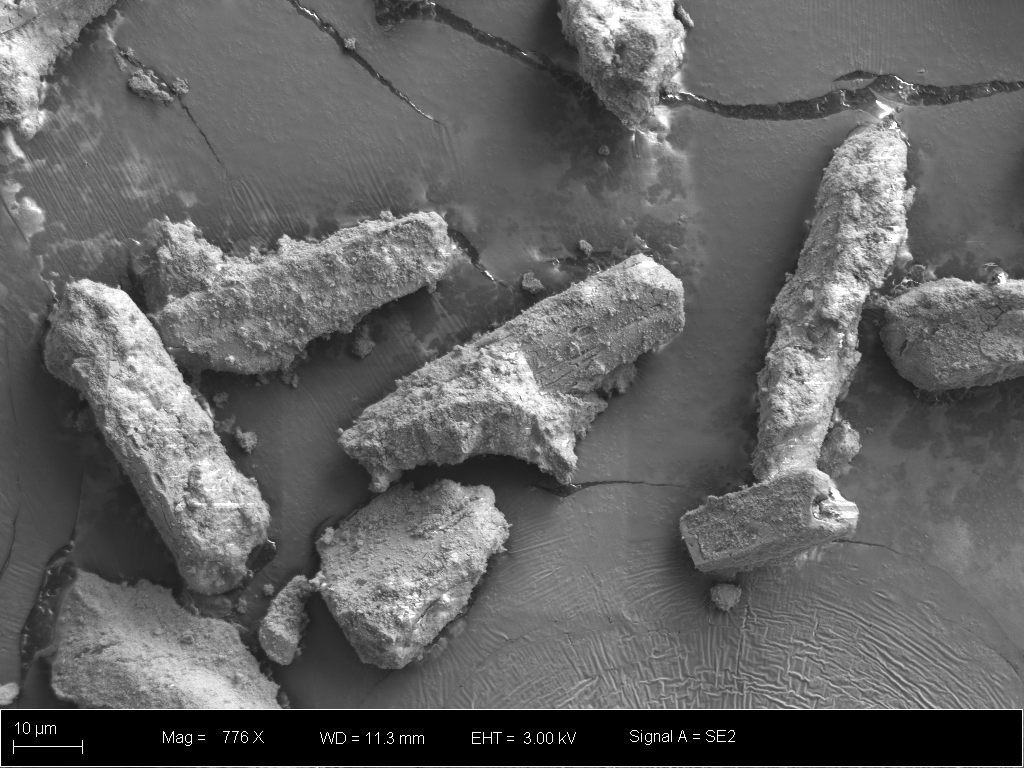


b


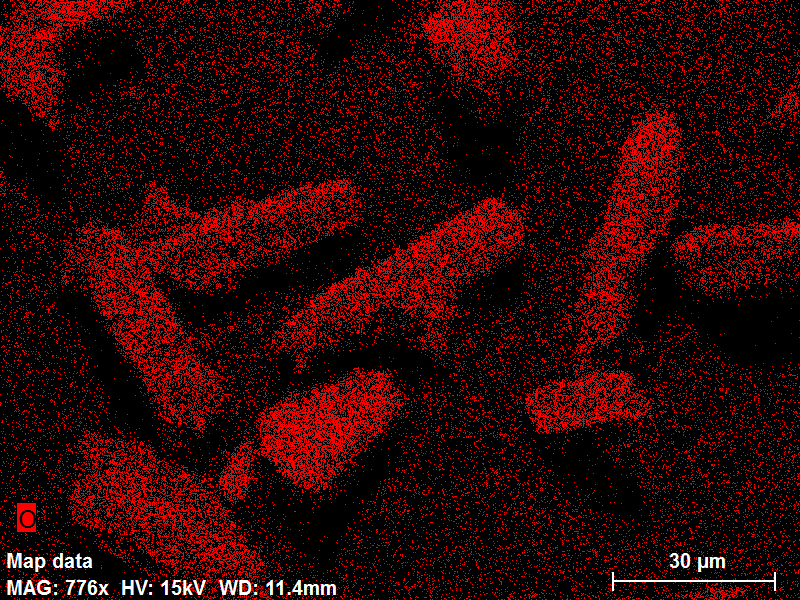


c


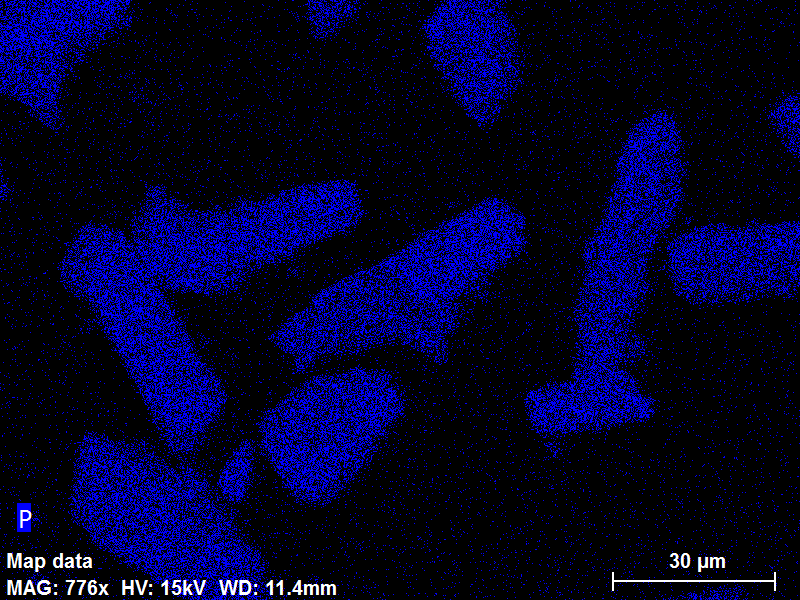


d


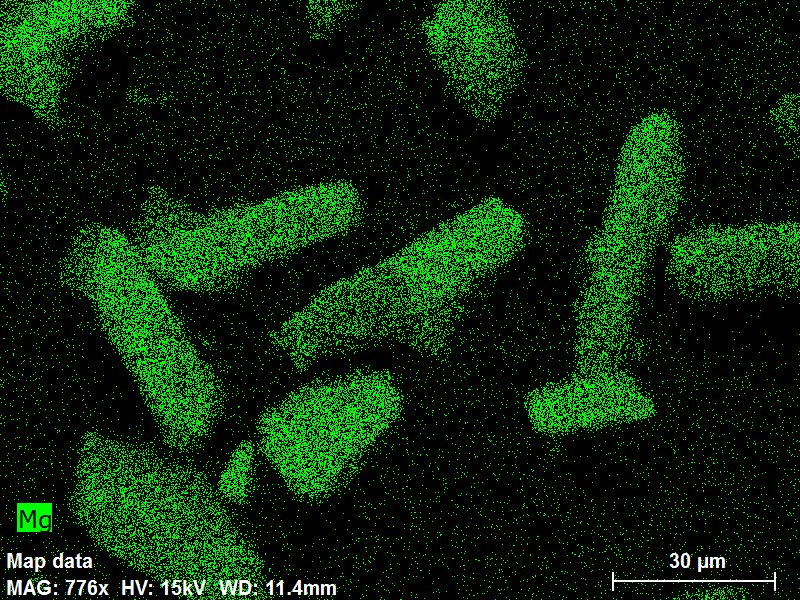


**Fig.S4** SEM-EDX analysis of struvite precipitate (a) SEM analysis (b) O element mapping analysis (c) P element mapping analysis (d) Mg element mapping analysis


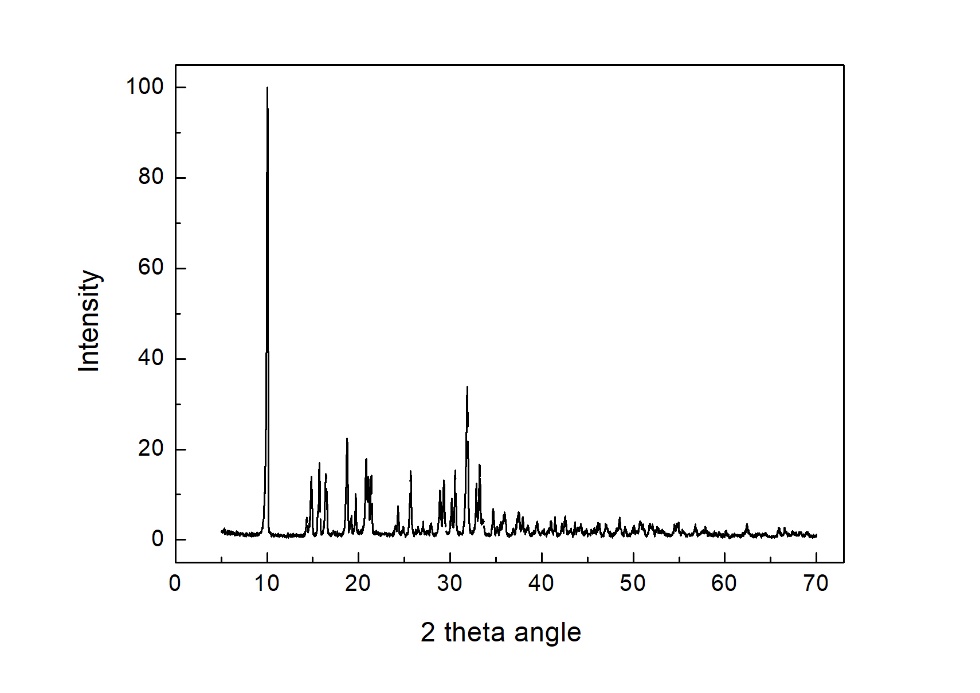


**Fig.S5** XRD analysis of struvite precipitate

**Table S1** Struvite crystallization modeling designed by RSM and the actual and prediction phosphorus recovery efficiency

| No. | X_1_-pH | X_2_-Mg/P | X_3_-rotate speed (rpm) | Y-Phosphorus removal efficiency | | |
| --- | --- | --- | --- | --- | --- | --- |
|  |  |  |  | Experimental recovery | Prediction recovery | |
| 1 | 7.5 | 1.0 | 150 | 53 | | 46 |
| 2 | 12.5 | 1.0 | 450 | 59 | 44 | |
| 3 | 7.5 | 1.8 | 450 | 55 | 59 | |
| 4 | 10.0 | 1.4 | 300 | 62 | 59 | |
| 5 | 12.5 | 1.8 | 150 | 25 | 17 | |
| 6 | 10.0 | 1.4 | 300 | 61 | 47 | |
| 7 | 12.5 | 1.8 | 450 | 36 | 41 | |
| 8 | 7.5 | 1.8 | 150 | 76 | 73 | |
| 9 | 10.0 | 1.4 | 300 | 12 | 10 | |
| 10 | 12.5 | 1.0 | 150 | 21 | 36 | |
| 11 | 7.5 | 1.0 | 450 | 15 | 36 | |
| 12 | 10.0 | 1.4 | 300 | 76 | 68 | |
| 13 | 5.9 | 1.4 | 300 | 75 | 82 | |
| 14 | 10.0 | 1.4 | 545 | 63 | 69 | |
| 15 | 10.0 | 0.7 | 300 | 85 | 83 | |
| 16 | 10.0 | 1.4 | 300 | 85 | 83 | |
| 17 | 14.0 | 1.4 | 300 | 79 | 83 | |
| 18 | 10.0 | 2.1 | 300 | 86 | 83 | |
| 19 | 10.0 | 1.4 | 55 | 77 | 83 | |
| 20 | 10.0 | 1.4 | 300 | 86 | 83 | |

**Table S2** ANOVA for response surface quadratic Model for phosphorus recovery efficiency

| Item | quadratic sum | degree of freedom | mean square error | F value | P value  (Prob>F) |
| --- | --- | --- | --- | --- | --- |
| Model | 1.03 | 9 | 0.11 | 7.89 | 0.0017 |
| X_1_ (pH) | 0.080 | 1 | 0.080 | 5.47 | 0.0414 |
| X_2_ (Mg/P) | 0.13 | 1 | 0.13 | 8.82 | 0.0141 |
| X_3_ (rotate speed) | 0.019 | 1 | 0.019 | 1.32 | 0.2774 |
| X_1_×X_2_ | 0.0002496 | 1 | 0.0002496 | 0.017 | 0.8984 |
| X_1_×X_3_ | 0.050 | 1 | 0.050 | 3.40 | 0.0948 |
| X_2_×X_3_ | 0.005715 | 1 | 0.005715 | 0.39 | 0.5448 |
| X_1_×X_1_ | 0.64 | 1 | 0.64 | 43.92 | <0.0001 |
| X_2_×X_2_ | 0.17 | 1 | 0.17 | 11.64 | 0.0066 |
| X_3_×X_3_ | 0.009284 | 1 | 0.009284 | 0.64 | 0.4429 |
| residual error | 0.15 | 10 | 0.015 |  |  |
| Cor Total | 1.18 | 19 |  |  |  |
| R^2^ | 0.8766 |  |  |  |  |

**Table S3** The percentage of elements in biochar and HFO/biochar

| Samples | C | N | O | P | Cl | Fe | Si | Na |
| --- | --- | --- | --- | --- | --- | --- | --- | --- |
| biochar | 84.80% | 1.17% | 12.15% | 0.44% | 1.44% |  |  |  |
| HFO/biochar | 48.07% | 0.68% | 31.28% | - | 0.93% | 17.33% | 0.34% | 1.38 |
